# Supplementary material for: Arginine GlcNAcylation of Rab small GTPases by the pathogen Salmonella Typhimurium
Source: Commun Biol. 2020 Jun 5;3:287. doi: 10.1038/s42003-020-1005-2 (PMC7275070; doi:10.1038/s42003-020-1005-2)
Supplement: Supplementary file 1 — Supplementary Information [file 42003_2020_1005_MOESM1_ESM.pdf]

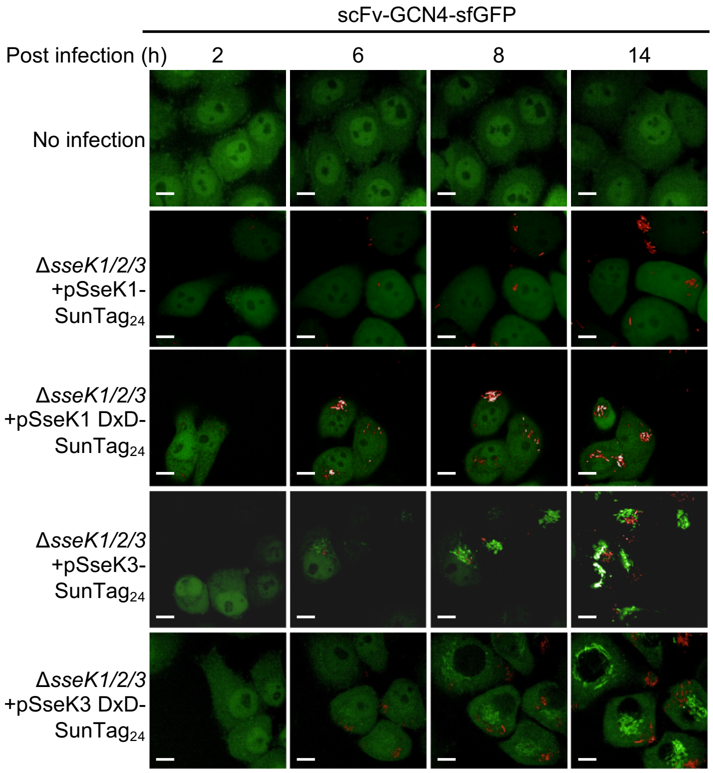

Supplementary Figure 1. Golgi-localization of T3SS-translocated SseK1 and SseK3. Subcellular localization of T3SS-translocated SseK1 and SseK3. HeLa cells stably expressing scFv-GCN4-GFP were infected with *S.* Typhimurium Δ*sseK1/2/3* complemented with a plasmid expressing mRFP together with a plasmid expressing the indicated SunTag24-tagged proteins. Mock (no infection) cells were set as a negative control. Shown are fluorescence images taken at the 14 h post infection. Scale bar, 10 μm.

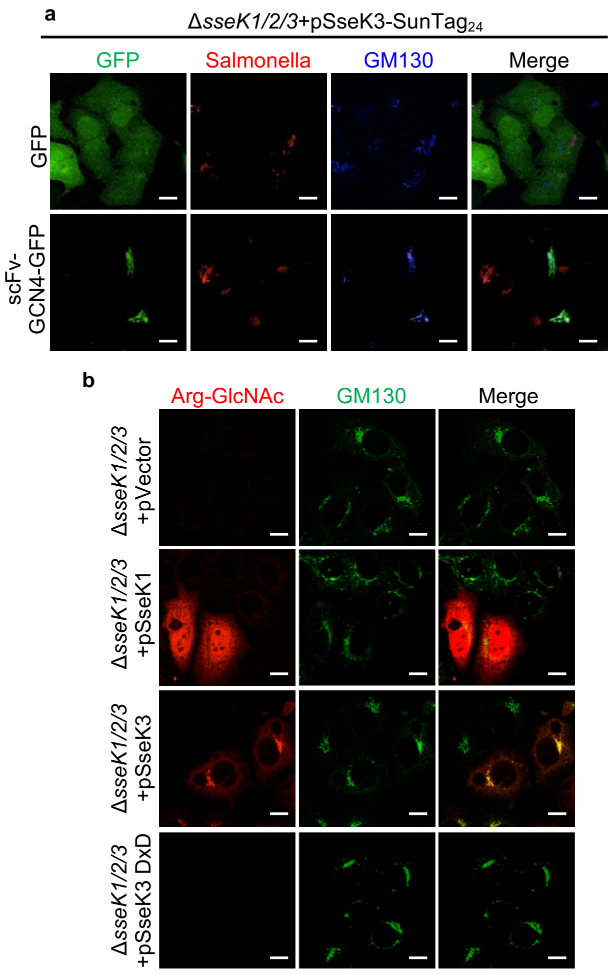

Supplementary Figure 2. Subcellular localization of SseK1/3 and their Arg-GlcNAcylated proteins during *Salmonella* infection. a, Golgi-localization of T3SS-translocated SseK3. HeLa cells stably expressing scFv-GCN4-GFP or GFP alone were infected with *S.* Typhimurium Δ*sseK1/2/3* complemented with a plasmid expressing mRFP together with a plasmid expressing SseK3-SunTag$_{24}$. Bacteria and Golgi apparatus were stained by anti-*Salmonella* antibody (red) and anti-GM130 antibody (blue), respectively. Shown are fluorescence images taken at 14 h post infection. **b**, HeLa cells were infected with *S.* Typhimurium Δ*sseK1/2/3* complemented with empty vector, SseK1- or SseK3-expressing plasmid as indicated. Shown are immunofluorescence detection of Arg-GlcNAcylation (red) and Golgi apparatus (green) at 14 h post infection. Data are representative from three independent experiments. Scale bar, 10 μm.

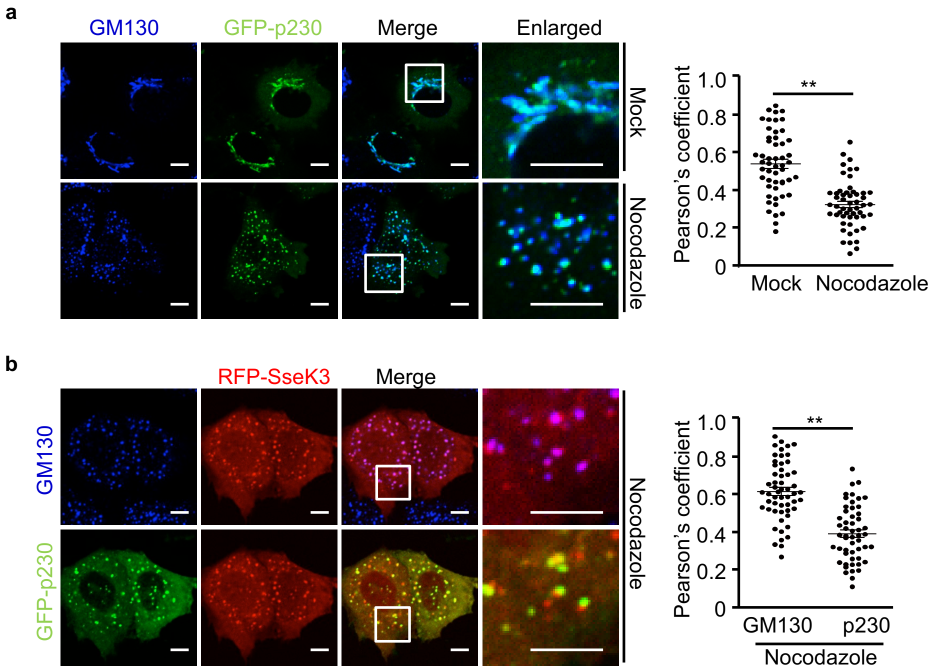

Supplementary Figure 3. *cis*-Golgi localization of transfected SseK3 in HeLa cells. a, Effects of Nocodazole on colocalization of *cis*- and *trans*-Golgi structures. HeLa cells were transfected with GFP-p230, treated with or without Nocodazole, and stained with anti-GM130 antibody. Colocalization of p230 and GM130 is shown in fluorescence images (left) and the statistics of Pearson correlation coefficient (right). **b**, RFP-SseK3- and GFP-p230-transfected HeLa cells were treated with Nocodazole for 1 h, and then subjected to immunofluorescence staining with the anti-GM130 antibody. Colocalization of SseK3 with GM130 or p230 is shown in florescence images (left) and the statistics of Pearson correlation coefficient (right). The Pearson correlation coefficient was calculated with more than 50 dots for each experiment by applying the Image J software (http://rsb.info.nih.gov/ij/). Vertical lines represent SEM. $**P<0.01$. Data are representative from three independent experiments. Scale bar, 10 μm.

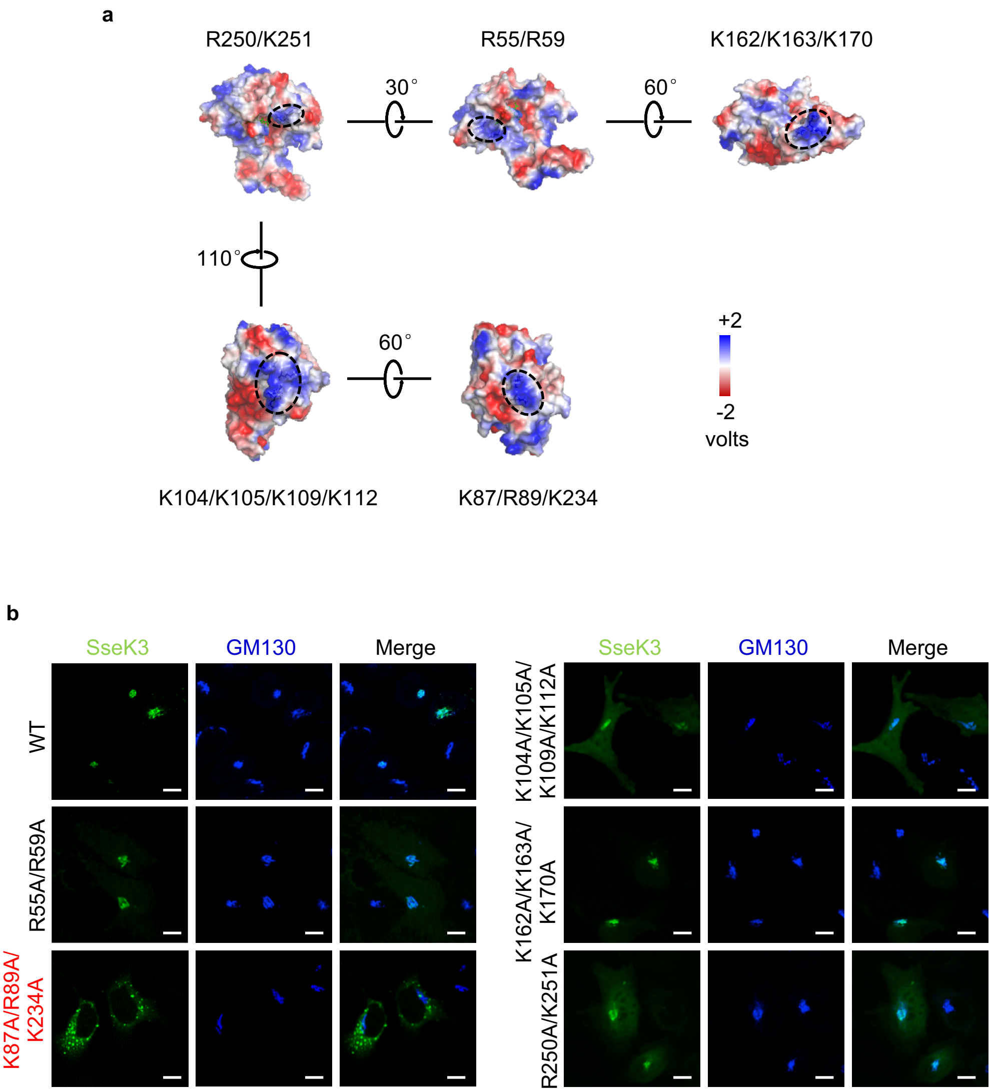
a
R250/K251
R55/R59
K162/K163/K170
30°
60°
110°
60°
+2
-2
volts
K104/K105/K109/K112
K87/R89/K234
b
SseK3
GM130
Merge
WT
R55A/R59A
K87A/R89A/
K234A
K104A/K105A/
K109A/K112A
K162A/K163A/
K170A
R250A/K251A

Supplementary Figure 4. Effects of polybasic regions in SseK3 on its Golgi-localization. **a,** Electrostatic potential surface schemes of Ssek3 (PDB code:6EYT) from different perspectives**.** The coloration from red to blue represents negatively to positively charged regions. The polybasic patches are circled with the basic amino acid residues shown in sticks. Adjacent schemes are changed by rotating the indicated angle along X (horizontal line) or Y (vertical line) axis. All structure figures in **a** were prepared in PyMOL and the surface potential were calculated using the APBS electrostatics tools with default parameters. **b**, HeLa cells were transfected with GFP-tagged WT or mutated SseK3, and subjected to immunofluorescence staining using anti-GM130 antibody. Shown are immunofluorescence detections of SseK3 (green) and Golgi apparatus (blue). Data are representative from three independent experiments. Scale bar, 10 μm.

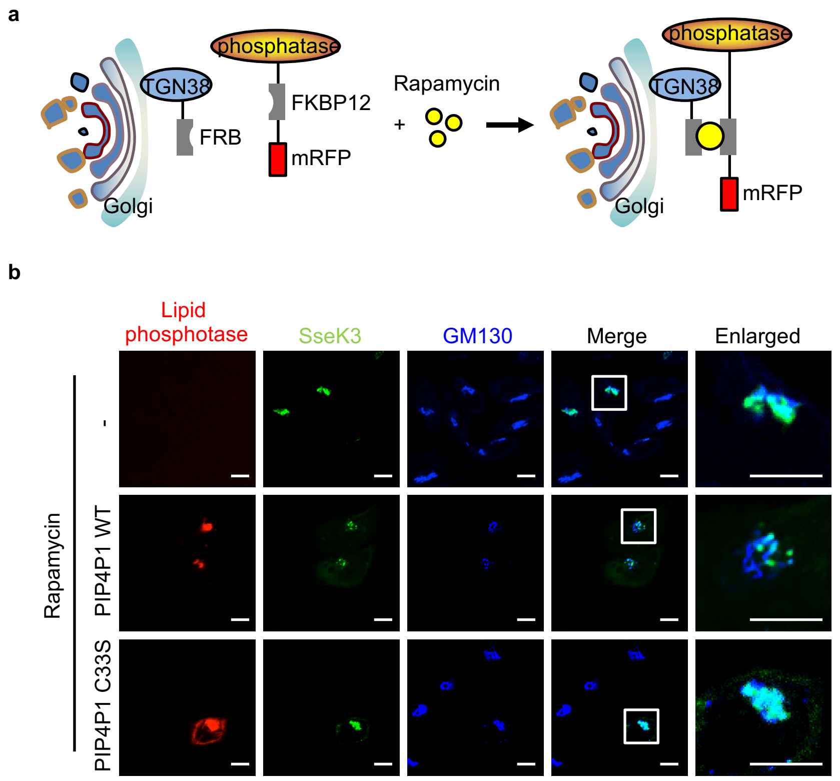

Supplementary Figure 5. Binding to PtdIns (4,5) P_2 is crucial for SseK3 localization on Golgi. a, Inducible recruitment system of phosphatases: rapamycin (1 μM) induces the heterodimerization of FRB and FKBP12, thereby recruiting the phosphatase to the Golgi structures to hydrolyze its target phospholipid. **b**, The effects of catalytic PtdIns (4,5) P_2 phosphatase translocation to the Golgi on the Golgi-localization of SseK3 in HeLa cells. Shown are immunofluorescence detection of phosphatases (red), Golgi apparatus (blue), and SseK3 (green). Data are representative from three independent experiments. Scale bar, 10 μm.

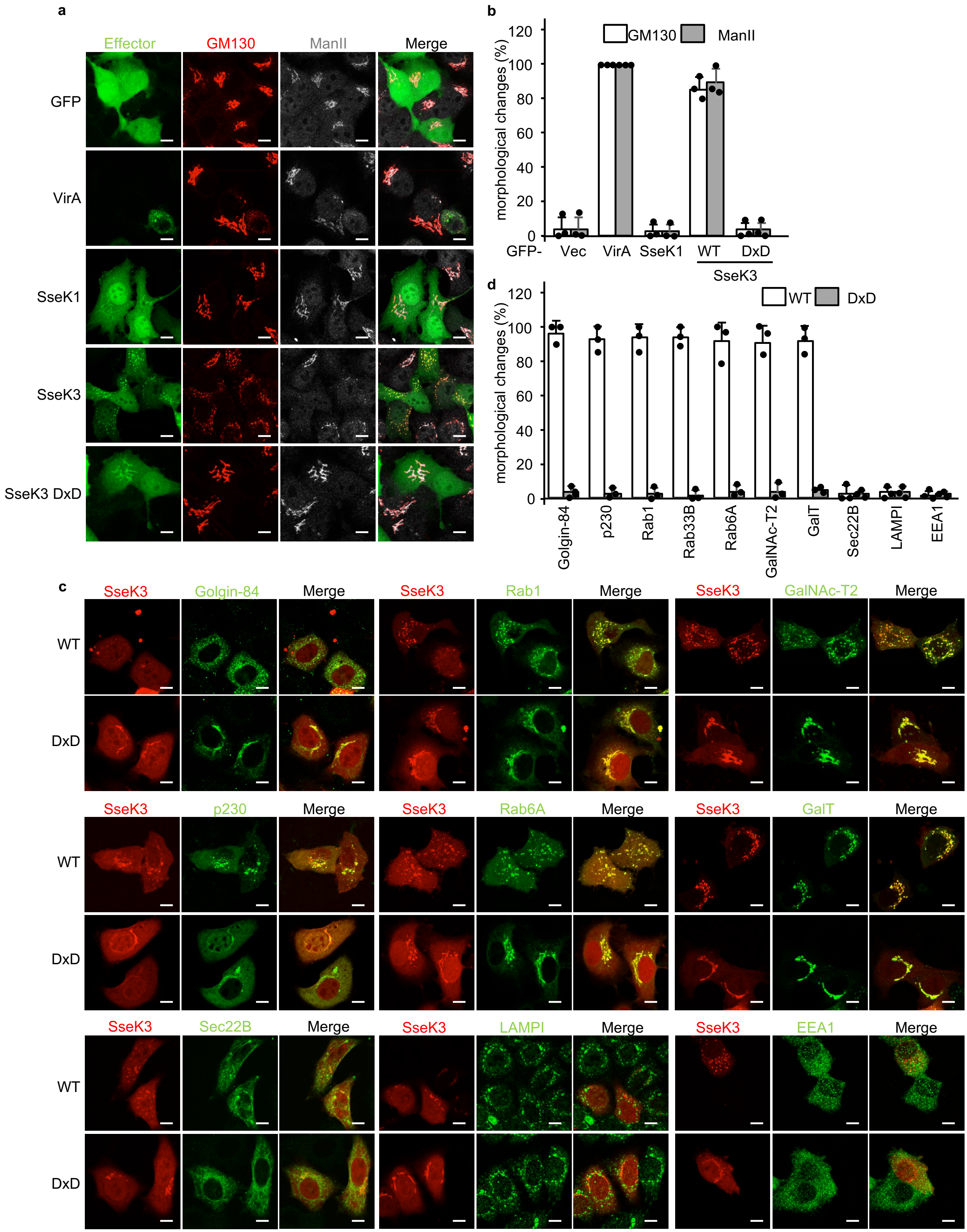
a
Effector
GM130
ManII
Merge
GFP
VirA
SseK1
SseK3
SseK3 DxD
b
GM130
ManII
morphological changes (%)
120
100
80
60
40
20
0
GFP-
Vec
VirA
SseK1
WT
DxD
SseK3
d
WT
DxD
morphological changes (%)
Golgin-84
p230
Rab1
Rab33B
Rab6A
GalNAc-T2
GalT
Sec22B
LAMPI
EEA1
c
SseK3
Golgin-84
Merge
Rab1
GalNAc-T2
WT
DxD
p230
Rab6A
GalT
Sec22B
LAMPI
EEA1

Supplementary Figure 6. Ectopic expression of SseK3 disrupts the Golgi structure. **a**,**b**, Effects of SseK3 transfection on endogenous Golgi protein immunostaining. **a**, HeLa cells were transfected with a plasmid expressing GFP alone or GFP-tagged indicated T3SS effectors. Shown are immunofluorescence staining using anti-GM130 and anti-ManII antibodies (Scale bar, 10 μm). **b**, Statistics of cells are listed according to **a**, and the percentages of Golgi disruption are mean ± SD from three determinations. At least 100 cells were counted for each experiment. **c**,**d**, SseK3 transfection disrupts the Golgi apparatus, but not ER, endosome or lysosome. **c**, RFP-tagged SseK3 or the SseK3 DxD mutant were transfected into HeLa cells together with indicated Golgi-located- (Golgi 84, p230, Rab1, Rab33B, Rab6A, GalNAc-T2 and GalT) or endoplasmic reticulum-located- (Sec22B) proteins, respectively. For LAMPI and EEA1, SseK3 transfected-cells were subjected to immunofluorescence staining using anti-LAMPI or anti-EEA1 antibody. **d**, Statistics of cells are listed according to **c**, and the percentages of organelle morphological changes are mean ± SD from three determinations. At least 100 cells were counted for each experiment. Scale bar, 10 μm. Data in **a** and **c** are representative from three independent experiments.

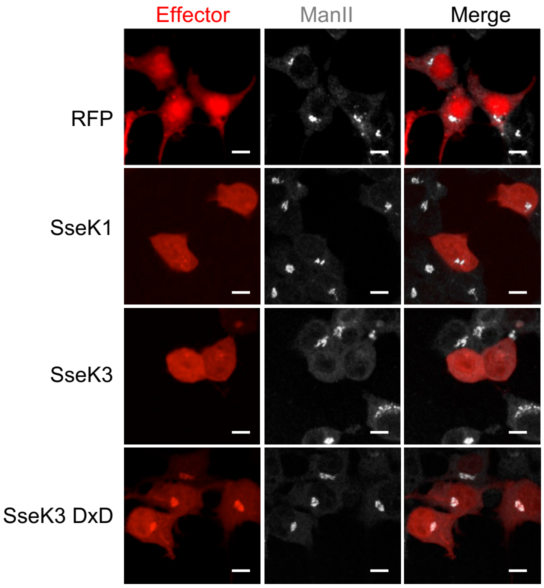

Supplementary Figure 7. Overexpression of SseK3 destroys the Golgi structure in 293T cells. 293T cells were transfected with a plasmid expressing RFP alone or RFP-tagged indicated T3SS effectors. The Golgi apparatus was stained by the anti-ManII antibody (grey). Data are representative from three independent experiments. (Scale bar, 10 μm).

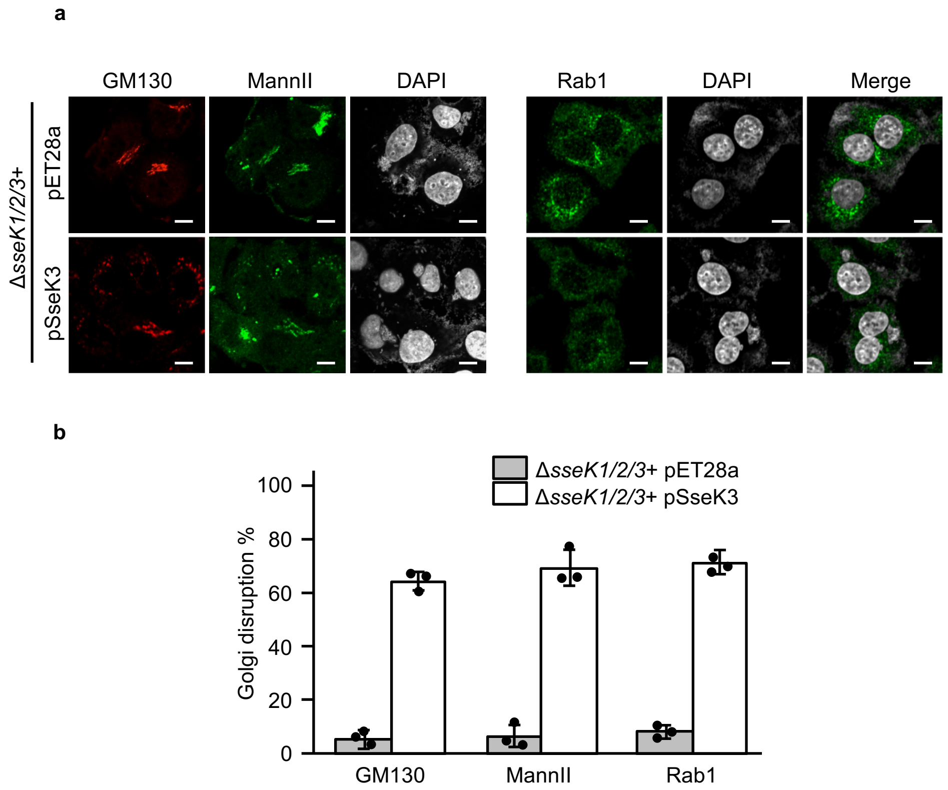

Supplementary Figure 8. SseK3 disturbed Golgi structure at 24 h post *Salmonella* infection. Effects of SseK3 on endogenous Golgi protein immunostaining during *Salmonella* infection. **a**, HeLa cells were infected with *S.* Typhimurium Δ*sseK1/2/3* complemented with an empty vector or a SseK3-expressing plasmid as indicated for 24 h. Shown are immunofluorescence staining using anti-GM130, anti-ManII or anti-Rab1A antibodies, and DAPI indicated the host and bacterial DNA. **b**, Statistics of cells are listed according to **a**, and the percentages of Golgi disruption are mean ± SD from three determinations. At least 100 cells were counted for each experiment. (Scale bar, 10 µm).

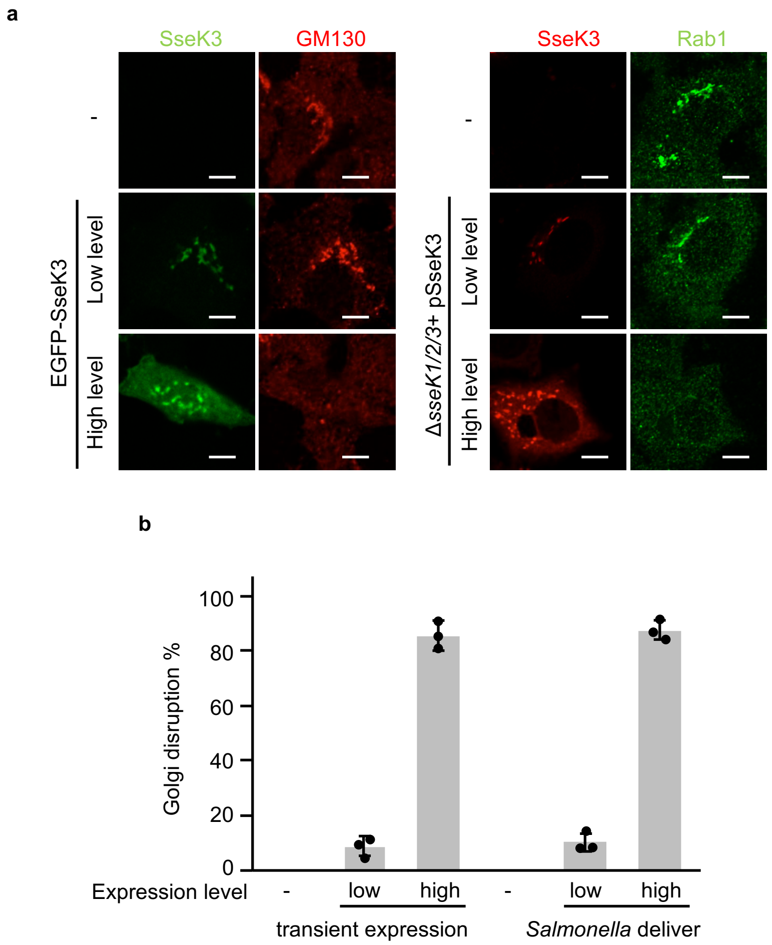

Supplementary Figure 9. Dose effects of SseK3 on the morphology of the Golgi structure. a, HeLa cells were transfected with a plasmid expressing GFP-SseK3 (left), or infected with *S.* Typhimurium Δ*sseK1/2/3* complemented with a SseK3-Flag-expressing plasmid for 24 h (right). Shown are immunofluorescence staining in cells expressing different SseK3 protein levels using anti-GM130 (left), anti-Rab1, or anti-Flag antibodies (right) in cells expressing different levels of SseK3 protein. **b**, Statistics of cells are listed according to **a**, and the percentages of Golgi disruption are mean ± SD from three determinations. At least 100 cells were counted for each experiment.

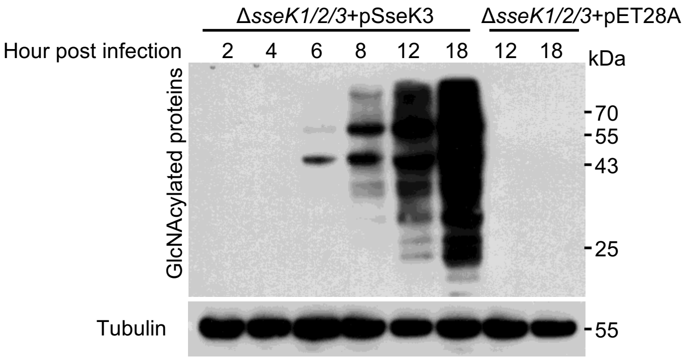

Supplementary Figure 10. Arginine GlcNAcylation pattern catalyzed by SseK3 during *Salmonella* infection. 293T cells were infected with *S.* Typhimurium Δ*sseK1/2/3* complemented with an empty vector or SseK3-expressing plasmid. Cell lysates were harvested at indicated time points. Shown are immunoblotting using anti-Arg-GlcNAc and anti-tubulin antibodies.

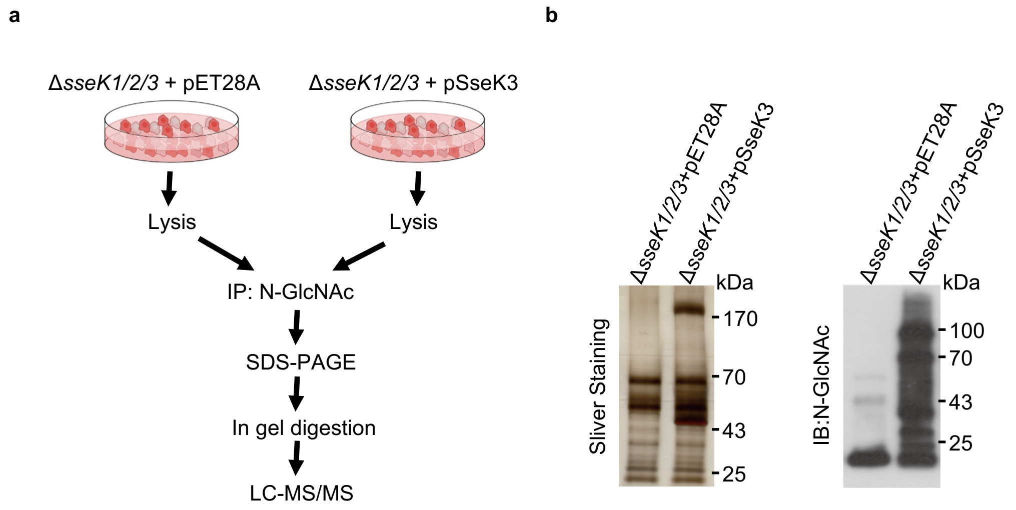

Supplementary Figure 11. Protein enrichment strategy to identify Arg-GlcNAcylated substrates of SseK3 during *S.* Typhimurium infection. a, A schematic diagram of the overall workflow that identifies potential host modified proteins by SseK3 during infection. HeLa cells were infected with *S.* Typhimurium Δ*sseK1/2/3* complemented with pET28A-SseK3 or pET28A vector, and then lysed prior to immunoprecipitation by the Arg-GlcNAc-specific antibody. The precipitates were further separated by SDS-PAGE before in-gel digestion with trypsin and LC-MS/MS analyses. **b**, Monitoring of the host Arg-GlcNAc-protein enrichment by silver staining analyses (left) and immunoblotting analyses (right).

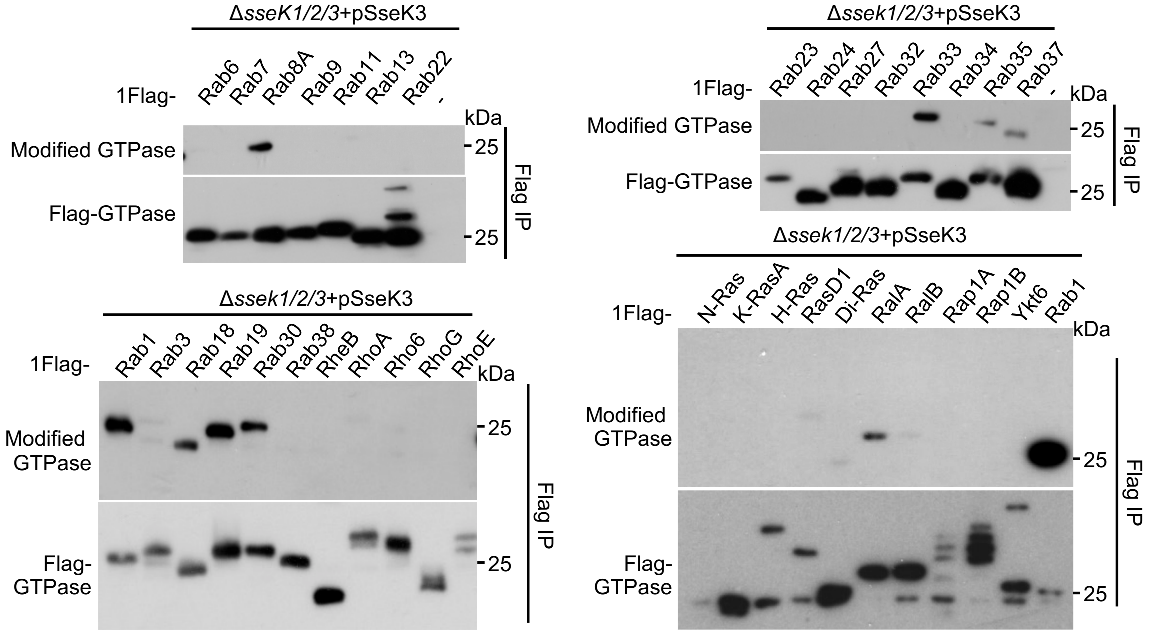

Supplementary Figure 12. Immunoblotting screen of Arg-GlcNAcylated Rab by SseK3 during infection. 293T cells were transfected with the indicated flag-tagged small GTPases expressing plasmids individually, and then infected with *S.* Typhimurium Δ*sseK1/2/3* complemented with pET28A-SseK3. Shown are immunoblots of anti-Flag immunoprecipitates (Flag IP).

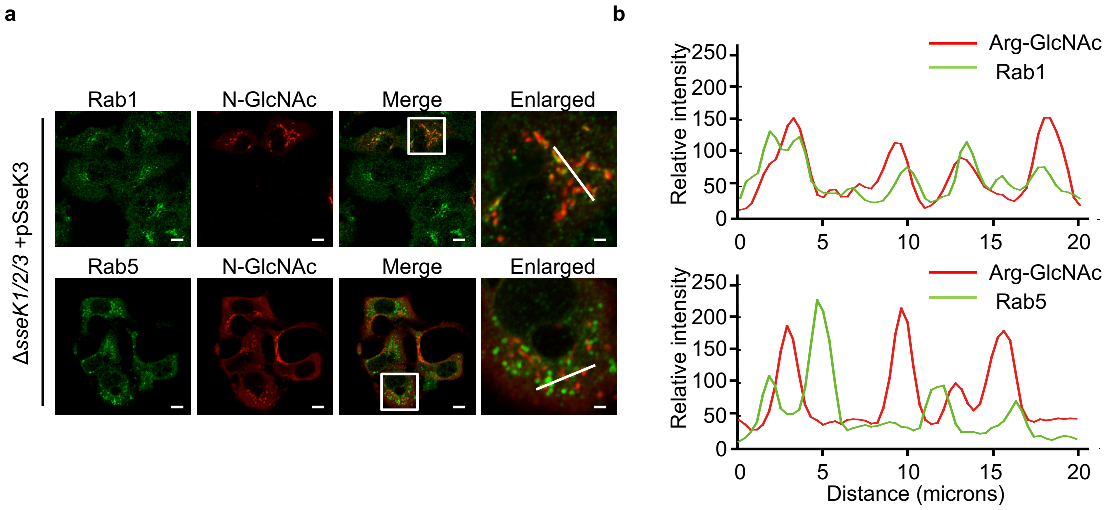

Supplementary Figure 13. Co-localization of arginine GlcNAc transferase activity of SseK3 with Rab1. a, HeLa cells were infected with *S.* Typhimurium Δ*sseK1/2/3* complemented with SseK3. Shown are immunofluorescence detections of Arg-GlcNAcylation (red) and Rab1 or Rab5 (green). Fluorescence images were taken at 18 h post infection (Scale bar, 10 μm). **b**, Linescan (right) was obtained from images in **a**. Data show the localization of Arg-GlcNActlated proteins relative to endogenous Rab1 (upper) and Rab5 (down). Linescan shows fluorescence intensity along a portion of the white line overlaying the image **a**.

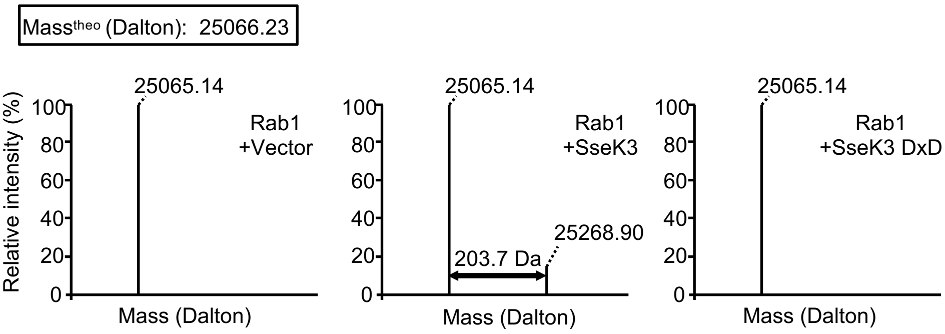

Supplementary Figure 14. Electrospray ionization (ESI)-mass spectrometry determination of the total mass of His-tagged Rab1 purified from vector, SseK3 or SseK3 DxD-expressed BL21 *E. coli* cells.

a

Δ*sseK1/2/3*+pSseK3 (WT)
Sequence Coverage: 62.43%
Matched peptides shown in **Bold Red**

```
  1 MSSMNPEYDY LFKLLLIGDS GVGKSCLLLR FADDTYTESY ISTIGVDFKI
 51 RTIELDGKTI KLQIWDTAGQ ERFRTITSSY YRGAHGIIVV YDVTDQESFN
101 NVKQWLQEID RYASENVNKL LVGNKCDLTT KKVVDYTTAK EFADSLGIPF
151 LETSAKNATN VEQSFMTMAA EIKKRMGPGA TAGGAEKSNV KIQSTPVKQS
201 GGGCC
```

Δ*sseK1/2/3*+pSseK3 (DxD)
Sequence Coverage: 61.95%
Matched peptides shown in **Bold Red**

```
  1 MSSMNPEYDY LFKLLLIGDS GVGKSCLLLR FADDTYTESY ISTIGVDFKI
 51 RTIELDGKTI KLQIWDTAGQ ERFRTITSSY YRGAHGIIVV YDVTDQESFN
101 NVKQWLQEID RYASENVNKL LVGNKCDLTT KKVVDYTTAK EFADSLGIPF
151 LETSAKNATN VEQSFMTMAA EIKKRMGPGA TAGGAEKSNV KIQSTPVKQS
201 GGGCC
```

b

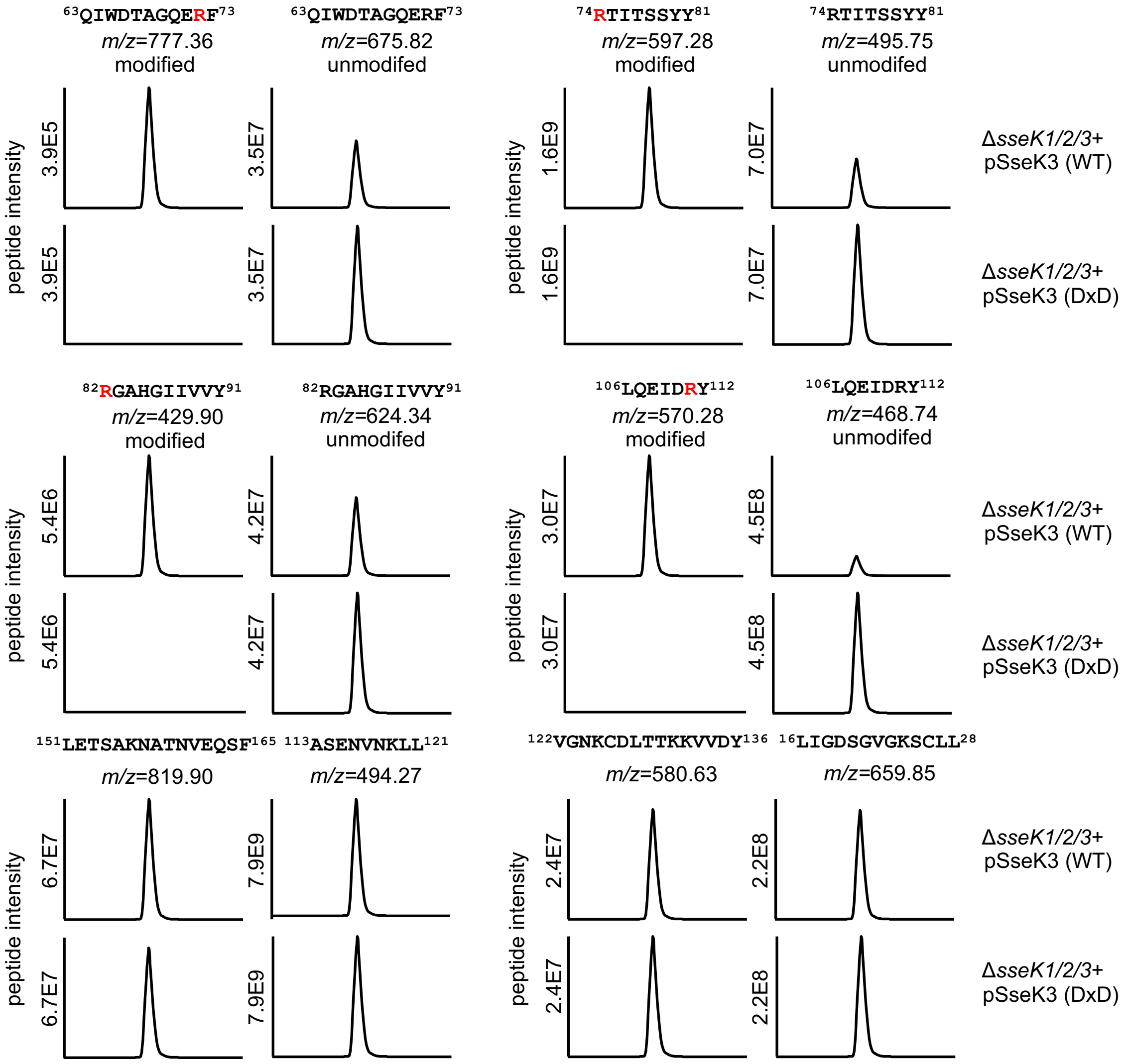

Supplementary Figure 15. MS detection of chymotrypsin digested Rab1 peptides. Rab1 stably expressed 293T cells were infected with *S.* Typhimurium Δ*sseK1/2/3* complemented with SseK3 or SseK3 DxD mutant. Immunoprecipitated Rab1 was then digested with chymotrypsin and analyzed by LC-MS. **a**, Detected Rab1 sequences are shown in red in LC-MS analyses and the GlcNAcylated peptide sequences are underlined. Asterisks indicate the modification sites. **b**, MS detection of Rab1 peptides that are covalently modified with one molecule of GlcNAc. Extracted ion chromatograms of peptides are shown with peak intensities indicating the relative amounts of peptides.

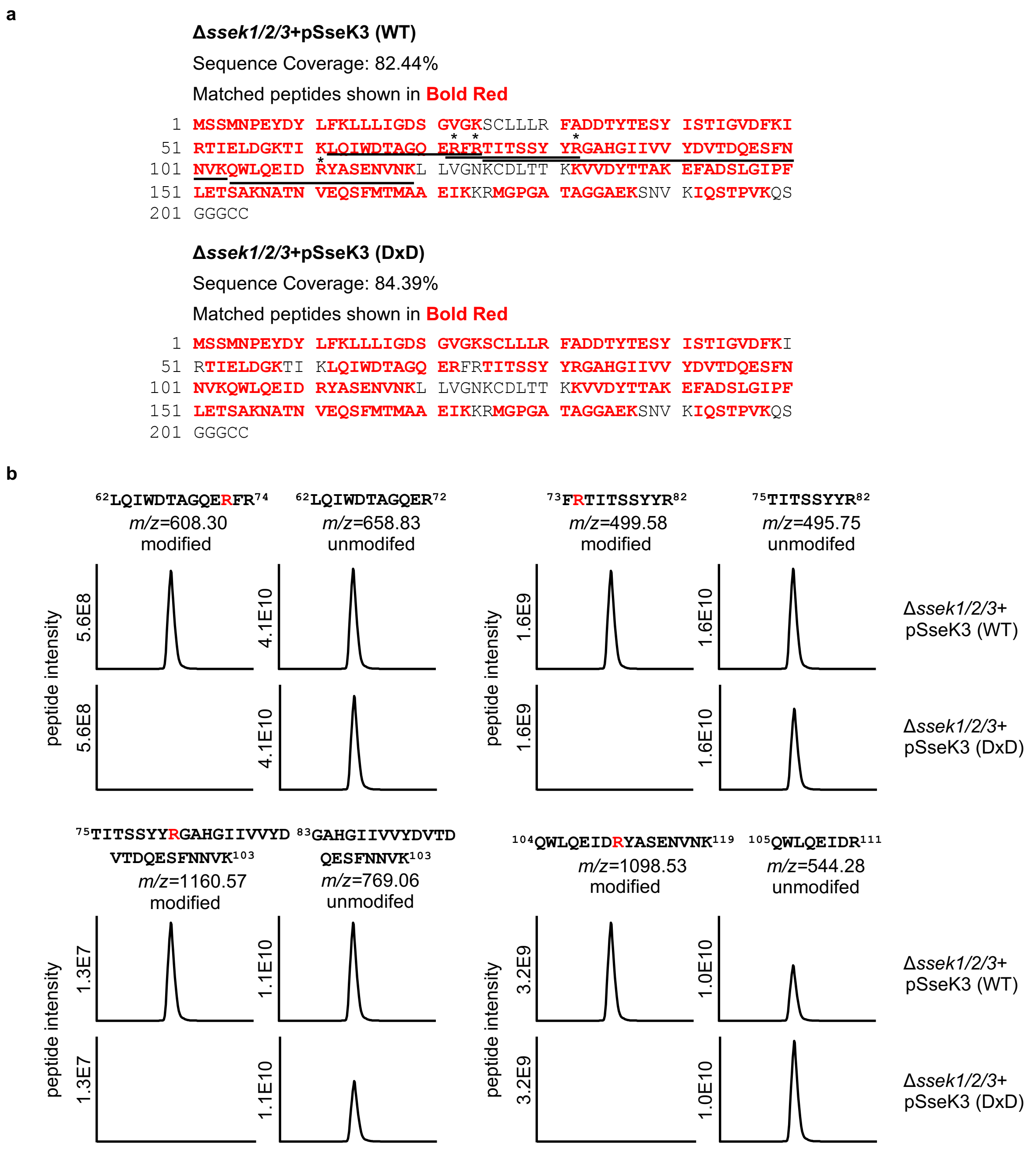

Supplementary Figure 16. MS detection of trypsin digested Rab1 peptides. Rab1 stably expressed 293T cells were infected with *S.* Typhimurium Δ*sseK1/2/3* complemented with SseK3 or SseK3 DxD mutant. Immunoprecipitated Rab1 was then digested with trypsin and analyzed by LC-MS. **a**, Detected Rab1 sequences are shown in red in LC-MS analyses and the GlcNAcylated peptide sequences are underlined. Asterisks indicate the modification sites. **b**, MS detection of Rab1 peptides that are covalently modified with one molecule of GlcNAc. Extracted ion chromatograms of peptides are shown with peak intensities indicating the relative amounts of peptides.

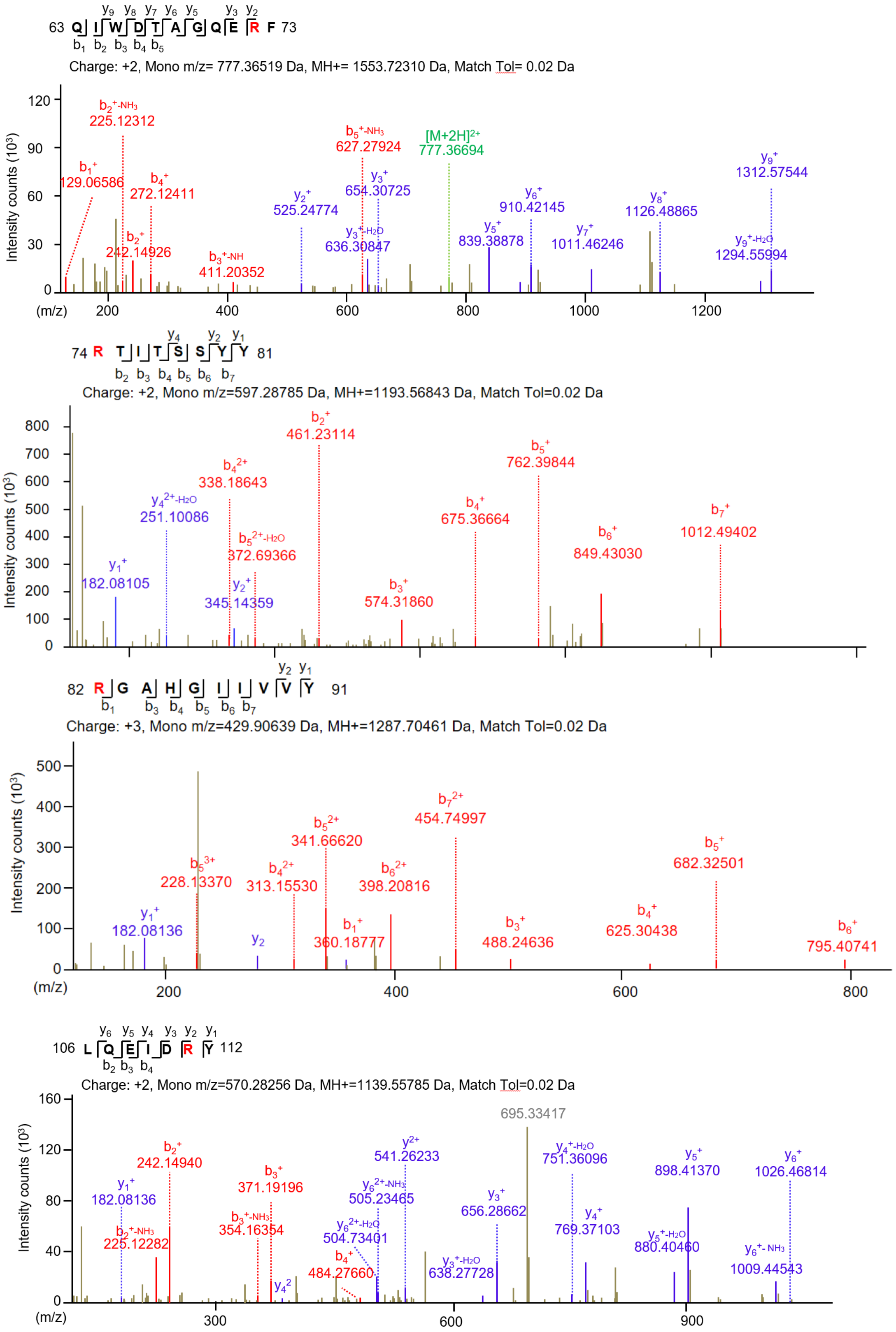

Supplementary Figure 17. Identification of the modification site of Rab1 from chymotrypsin digested Rab1 peptide. Rab1 stably expressed 293T cells were infected with *S.* Typhimurium Δ*sseK1/2/3* complemented with SseK3 or SseK3 DxD mutant. Immunoprecipitated Rab1 was then digested with chymotrypsin and analyzed by LC-MS/MS**.** The modification sites were determined by higher collisional dissociation (HCD) analysis in the Q Exactive Plus. The MS/MS spectrums of modified peptides are shown.

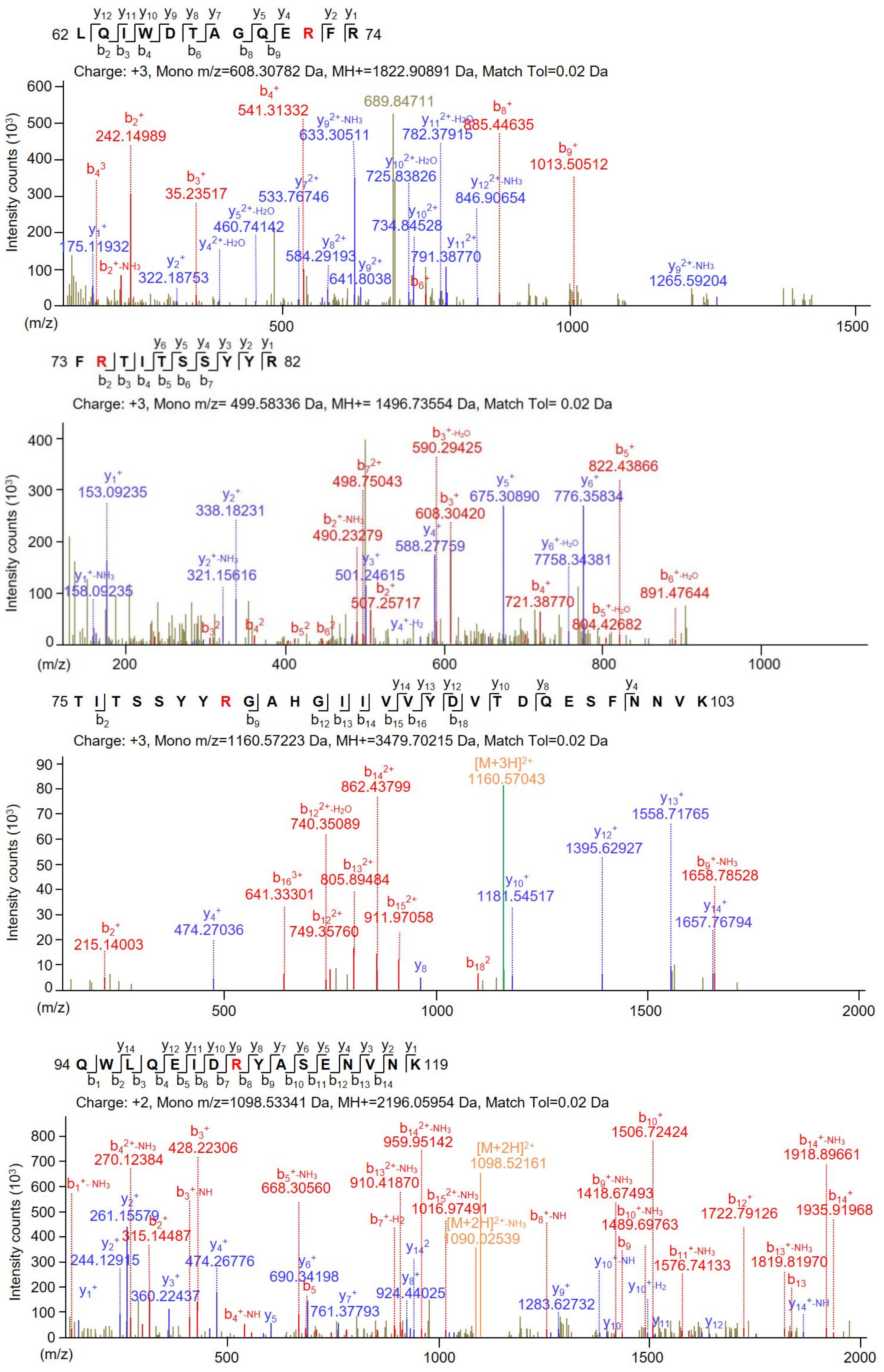

Supplementary Figure 18. Identification of the modification site of Rab1 from trypsin digested Rab1 peptide. Rab1 stably expressed 293T cells were infected with *S.* Typhimurium Δ*sseK1/2/3* complemented with SseK3 or SseK3 DxD mutant. Immunoprecipitated Rab1 was then digested with trypsin and analyzed by LC-MS/MS**.** The modification sites were determined by higher collisional dissociation (HCD) analysis in the Q Exactive Plus. The MS/MS spectrums of modified peptides are shown.

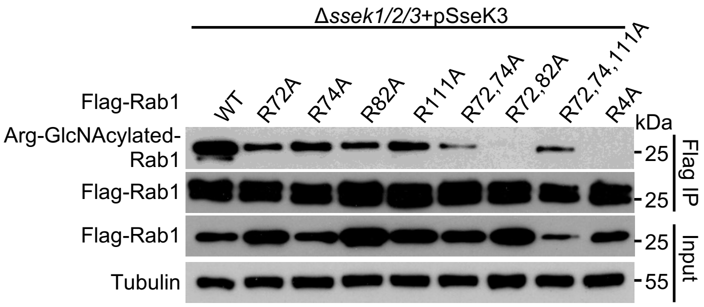

Supplementary Figure 19. Verification of the modification sites of Rab1 by immunoblotting. 293T cells were transfected with the indicated flag-tagged Rab1 wildtype or mutants, then infected with *S.* Typhimurium Δ*sseK1/2/3* complemented with pET28A-SseK3. Shown are immunoblots of input and immunoprecipitates (Flag IP) with the indicated antibodies. Data are representative from three independent experiments.

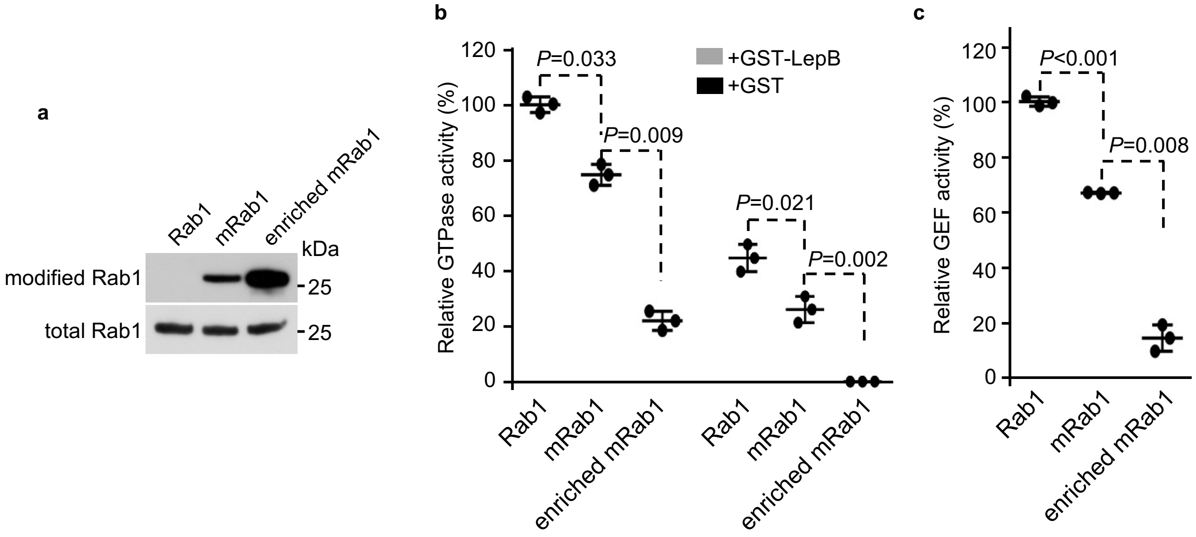

Supplementary Figure 20. Argnine GlcNAcylation of Rab1 attenuates the GTPase activity of Rab1 and GEF Activity toward Rab1. His-tagged Rab1 was purified from vector- or SseK3-expressed BL21 *E. coli* cells. Modified-Rab1 (mRab1) was subjected to further enrichment using an anti-Arg-GlcNAc antibody. **a**, Monitoring of the Arg-GlcNAc-Rab1 enrichment by immunoblotting analyses. **b**, **c**, Unmodified Rab1, modified Rab1 (mRab1), or enriched mRab1 was subjected to GTPase activity assay with or without the addition of recombinant LepB (**b**), or subjected to the GEF assay with the addition of recombinant DrrA (**c**). This data corresponds to Fig. 5a,b. The percentages of relative activity are mean ± SD from three experiments.

**P<0.01.

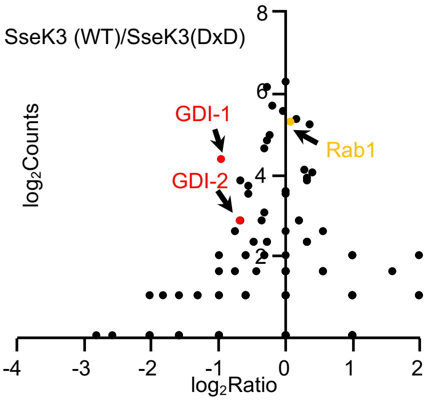

Supplementary Figure 21. Quantification of Rab1-binding proteins immunoprecipitated from 293T cells. Scatter plots of protein ratios as a function of their relative abundance (denoted by MS/MS spectral counts). The ratio was calculated as spectral counts in SseK3 transfected samples divided by those in SseK3 DxD transfected samples. Small ratios indicate decreased binding efficiency with Rab1 in SseK3 treated cells. Red dots correspond to GDI-1 and GDI-2, and the yellow dot corresponds to immunoprecipitated Rab1. This data corresponds to Fig. 5c.

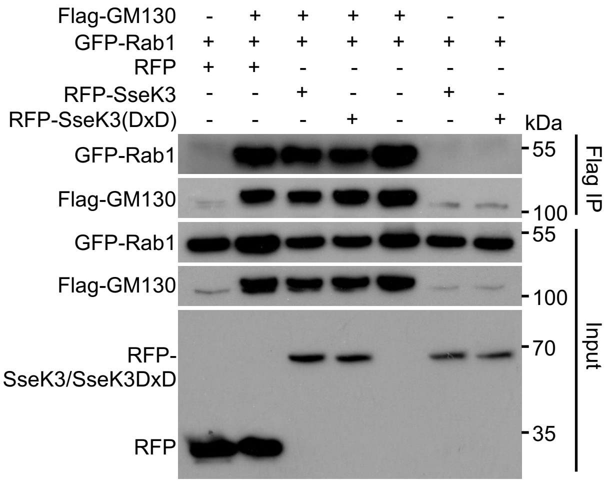

Supplementary Figure 22. Effects of SseK3 on the interaction of Rab1 and GM130. Coimmunoprecipitation assay of GFP-Rab1 with Flag-GM130 expressed in 293T cells in the presence or in the absence of RFP-SseK3 (WT or DxD). Data are representatives from three independent experiments.

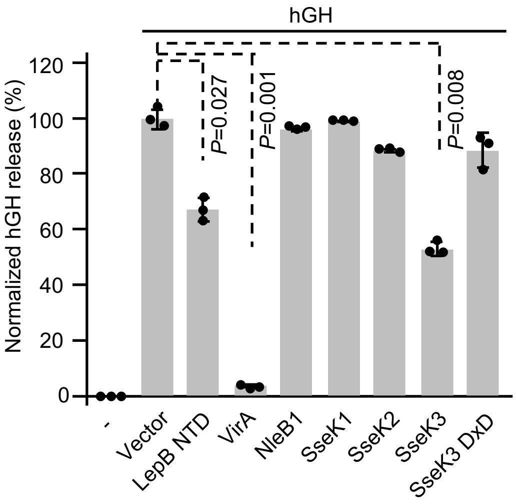

Supplementary Figure 23. Effects of SseK expression on hGH secretion. 293T cells were co-transfected with a plamsid expressing 4×FKBP-hGH and the indicated GFP-tagged effectors plasmids for 18 h. Drug-induced hGH release was quantified by ELISA and normalized to that of control cells expressing GFP alone. The percentages of relative released hGH are mean ± SD from three experiments.

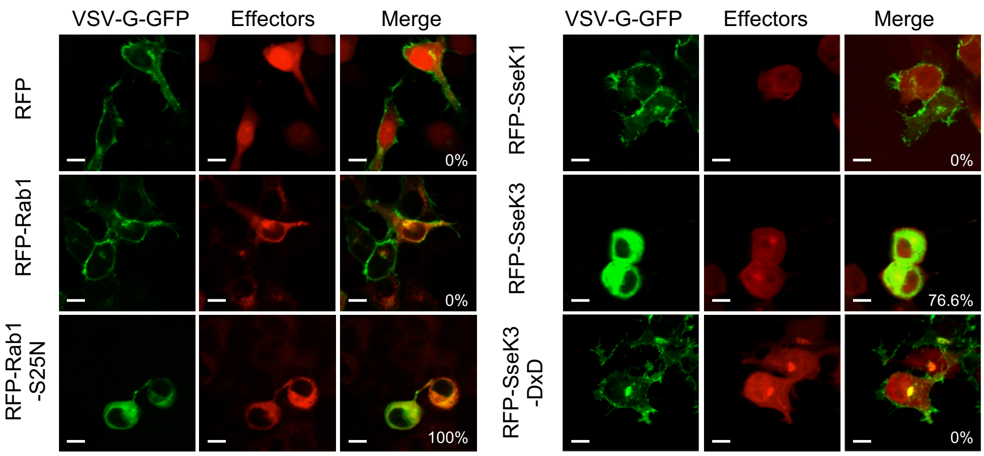

Supplementary Figure 24. SseK3 blocks VSV-G trafficking from ER to the plasma membrane in 293T cells. VSV-G-GFP-expressing 293T cells transfected with plasmids expressing indicated RFP-tagged-proteins were incubated at 40.5 °C for 16 h and then moved to 32 °C for 4 h. Confocal fluorescence images of VSV-G-GFP localization are shown. Scale bars, 10 µm. Statistics of cells showing VSV-G trafficking defects are listed in the corresponding fluorescence images (at least 100 cells were counted for each experiment).

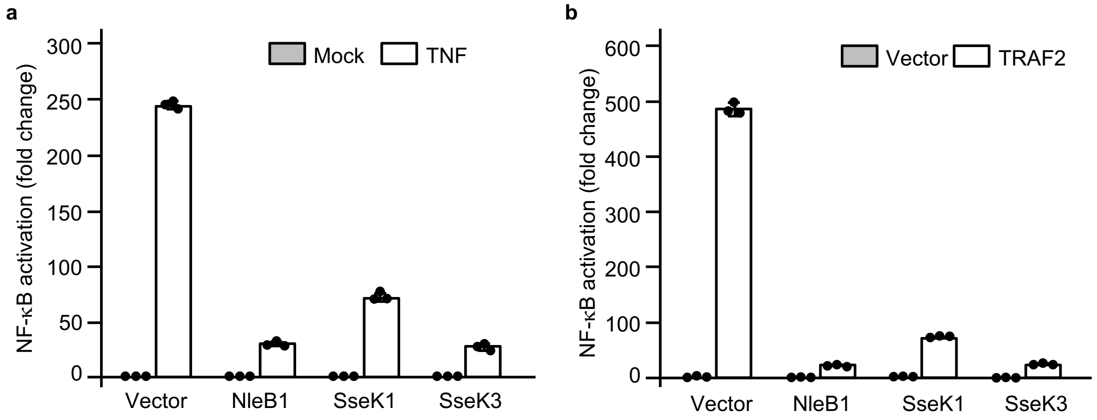

Supplementary Figure 25. Both SseK1 and SseK3 block the TNF signaling pathway. 293T cells were co-transfected with an NF-κB-dependent luciferase reporter plasmid, pTK-Renilla luciferase plasmid and the indicated pCS2-GFP-effector plasmids. The NF-κB pathway was activated overnight with 50 ng/ml TNFα (a), or overexpression of TRAF2 for 24 h (b), prior to cell lysis and analysis of luciferase activity. Results are presented as fold activation relative to unstimulated cells. Data shown are mean of three independent experiments ± SEM.

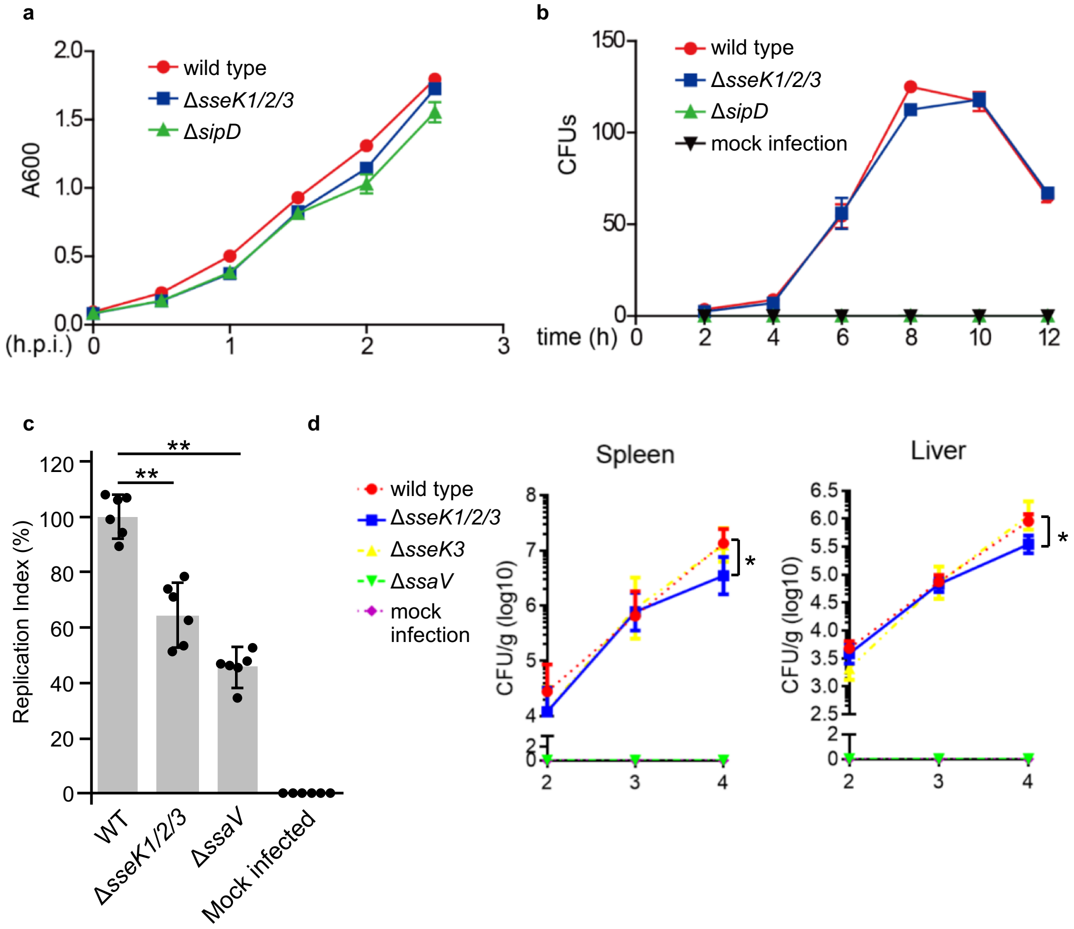

Supplementary Figure 26. Effects of SseK1/2/3 on bacterial replication *in vitro* and *in vivo*. **a**, *S.* Typhimurium dynamic growth curve in LB broth media; **b**, *S.* Typhimurium dynamic growth curve in HeLa cells; **c**, *S.* Typhimurium replication in macrophages RAW264.7 cells. Results shown are mean values ± SD from six independent experiments; **d**, *S.* Typhimurium dynamic growth in mice. A minimum of 5 mice was used for each group. Results shown are mean values ± SEM (error bar). *P<0.05, ***P*<0.01.
