# Supplementary material for: Arginine GlcNAcylation of Rab small GTPases by the pathogen Salmonella Typhimurium
Source: Commun Biol. 2020 Jun 5;3:287. doi: 10.1038/s42003-020-1005-2 (PMC7275070; doi:10.1038/s42003-020-1005-2)
Supplement: Supplementary file 2 — Description of Additional Supplementary Files [file 42003_2020_1005_MOESM2_ESM.pdf]

Description of Additional Supplementary Files

File Name: Supplementary Movie 1
Description: **Mock (no infection) cells were set as a negative control.**
HeLa cells stably expressing scFv-GCN4-GFP were mock infected. Shown are real-time videos of representative fields of the uninfected cells. The width of the field of view is 202.4 µm. The time-lapse images are in Figure 1a. (MP4 3031 KB)

File Name: Supplementary Movie 2
Description: **Subcellular localization of T3SS-translocated SseK1.**
HeLa cells stably expressing scFv-GCN4-GFP were infected with S. Typhimurium ΔsseK1/2/3 complemented with a plasmid expressing SseK1-SunTag24. Shown are real-time videos of representative fields of the infected cells. The width of the field of view is 202.4 µm. The time-lapse images are in Figure 1a. (MP4 2810 KB)

File Name: Supplementary Movie 3
Description: **Subcellular localization of T3SS-translocated SseK3.**
HeLa cells stably expressing scFv-GCN4-GFP were infected with S. Typhimurium ΔsseK1/2/3 complemented with a plasmid expressing SseK3-SunTag24. Shown are real-time videos of representative fields of the infected cells. The width of the field of view is 202.4 µm. The time-lapse images are in Figure 1a. (MP4 2742 KB)

File Name: Supplementary Data 1
Description: Uncropped blot images.

File Name: Supplementary Data 1
Description: All source data underlying the graphs and charts presented in the main figures.
