# Supplementary material for: Arginine GlcNAcylation of Rab small GTPases by the pathogen Salmonella Typhimurium
Source: Commun Biol. 2020 Jun 5;3:287. doi: 10.1038/s42003-020-1005-2 (PMC7275070; doi:10.1038/s42003-020-1005-2)
Supplement: Supplementary file 6 — Supplementary Data 1 [file 42003_2020_1005_MOESM6_ESM.pdf]

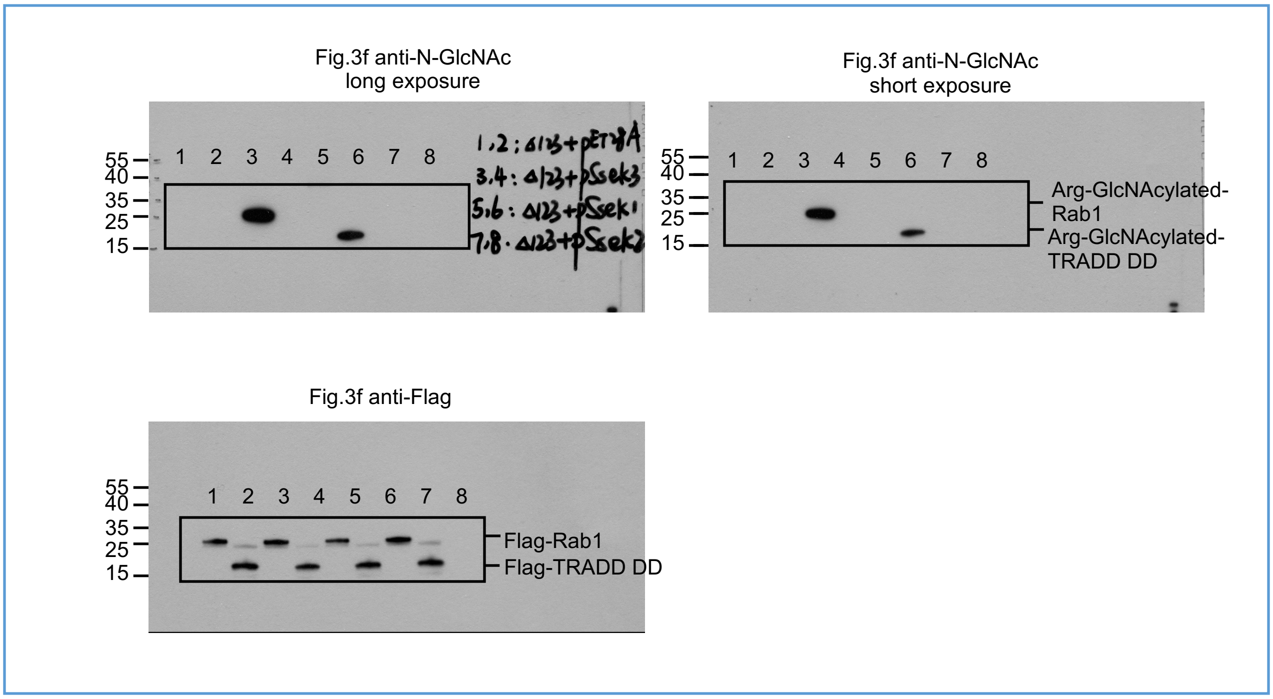

Fig.3f anti-N-GlcNAc long exposure
1 2 3 4 5 6 7 8
55 40 35 25 15
1,2: Δ123+pET28A
3,4: Δ123+pSsek3
5,6: Δ123+pSseK1
7,8: Δ123+pSsek2
Fig.3f anti-N-GlcNAc short exposure
Arg-GlcNAcylated-Rab1
Arg-GlcNAcylated-TRADD DD
Fig.3f anti-Flag
Flag-Rab1
Flag-TRADD DD

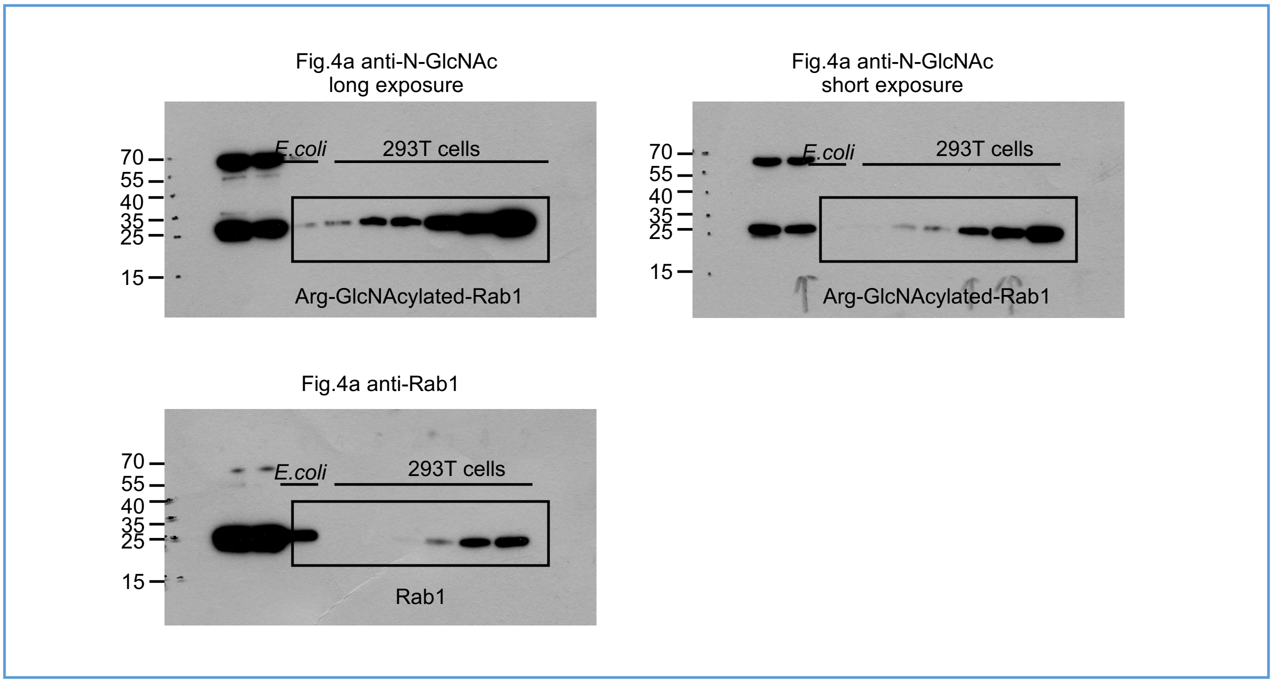

Fig.4a anti-N-GlcNAc long exposure
70 55 40 35 25 15
E.coli
293T cells
Arg-GlcNAcylated-Rab1
Fig.4a anti-N-GlcNAc short exposure
Fig.4a anti-Rab1
Rab1

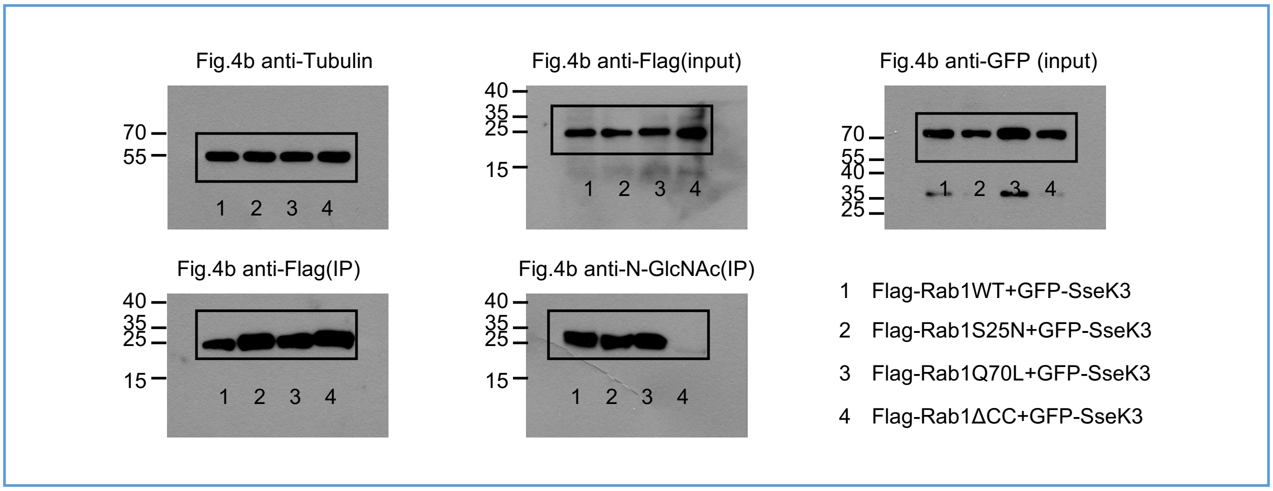

Fig.4b anti-Tubulin
Fig.4b anti-Flag(input)
Fig.4b anti-GFP (input)
Fig.4b anti-Flag(IP)
Fig.4b anti-N-GlcNAc(IP)
1 2 3 4
1 Flag-Rab1WT+GFP-SseK3
2 Flag-Rab1S25N+GFP-SseK3
3 Flag-Rab1Q70L+GFP-SseK3
4 Flag-Rab1ΔCC+GFP-SseK3

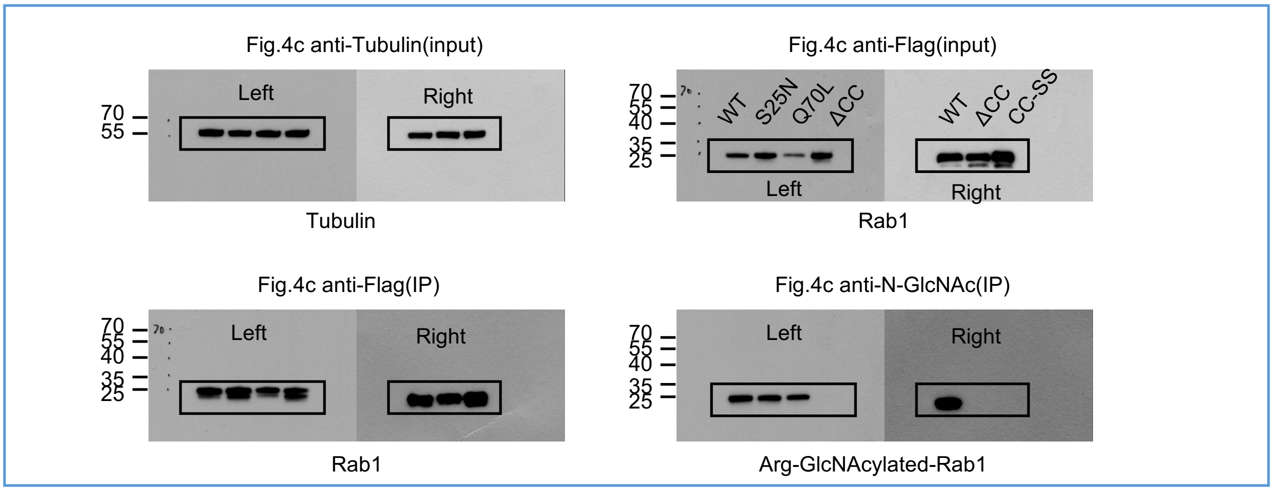
Fig.4c anti-Tubulin(input)
70
55
Left
Right
Tubulin
Fig.4c anti-Flag(input)
70
55
40
35
25
WT
S25N
Q70L
ΔCC
WT
ΔCC
CC-SS
Left
Right
Rab1
Fig.4c anti-Flag(IP)
70
55
40
35
25
Left
Right
Rab1
Fig.4c anti-N-GlcNAc(IP)
70
55
40
35
25
Left
Right
Arg-GlcNAcylated-Rab1

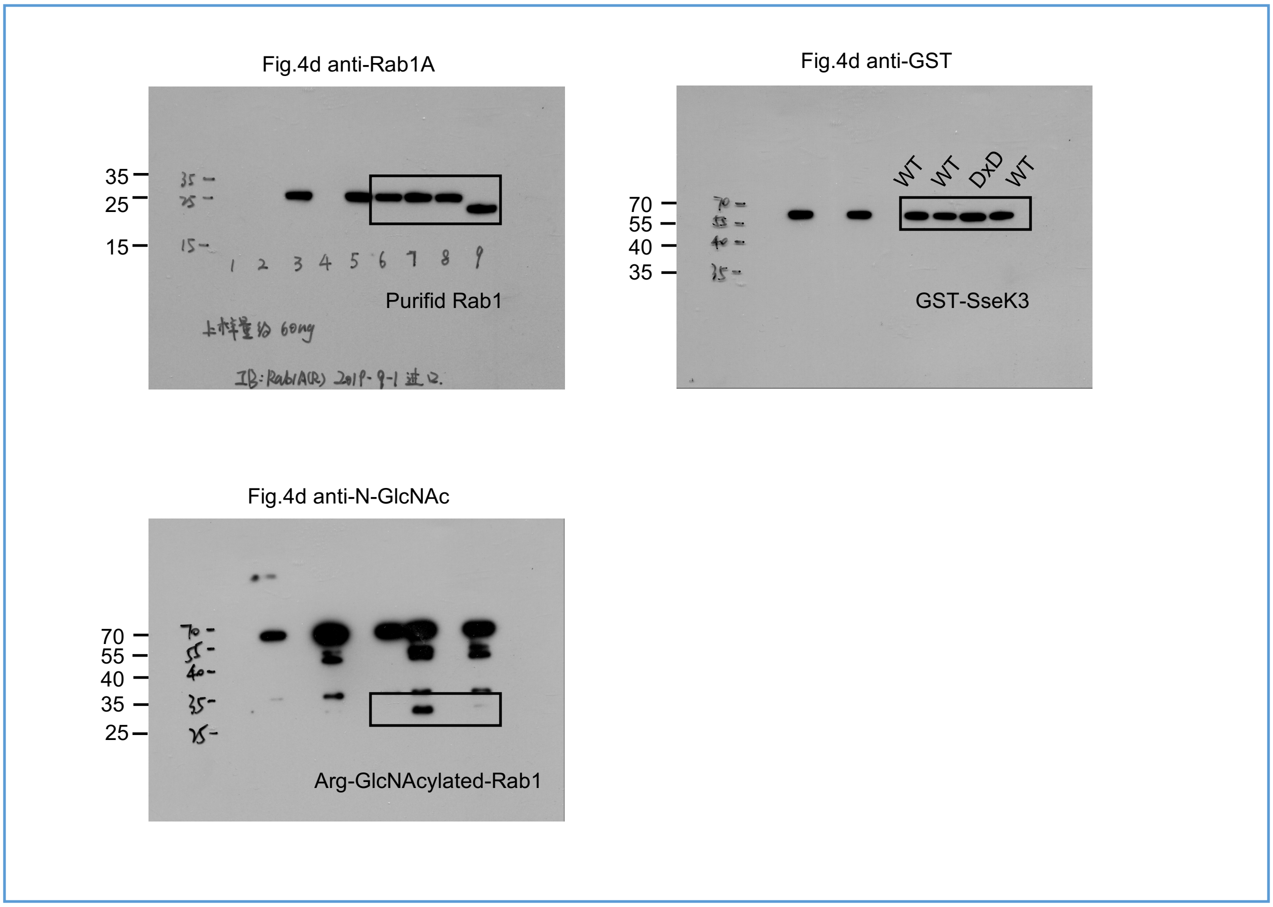
Fig.4d anti-Rab1A
35
25
15
1 2 3 4 5 6 7 8 9
Purifid Rab1
Fig.4d anti-GST
70
55
40
35
WT
WT
DxD
WT
GST-SseK3
Fig.4d anti-N-GlcNAc
70
55
40
35
25
Arg-GlcNAcylated-Rab1

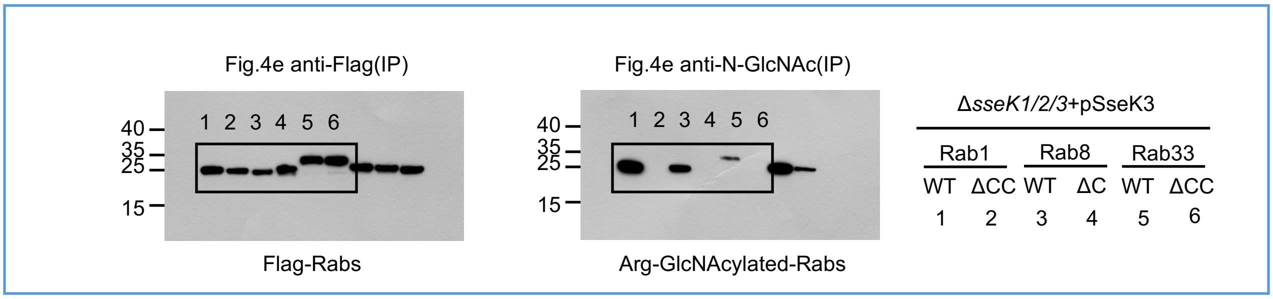
Fig.4e anti-Flag(IP)
40
35
25
15
1 2 3 4 5 6
Flag-Rabs
Fig.4e anti-N-GlcNAc(IP)
40
35
25
15
1 2 3 4 5 6
Arg-GlcNAcylated-Rabs
ΔsseK1/2/3+pSseK3
Rab1
Rab8
Rab33
WT ΔCC WT ΔC WT ΔCC
1 2 3 4 5 6

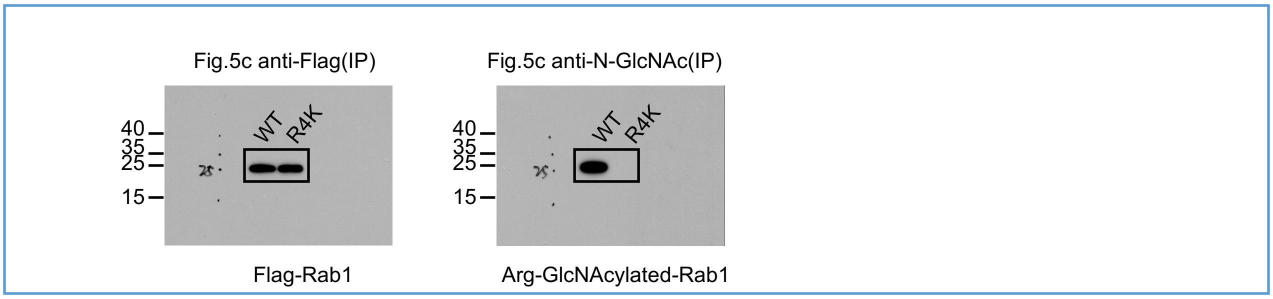
Fig.5c anti-Flag(IP)
Fig.5c anti-N-GlcNAc(IP)
40
35
25
15
WT
R4K
Flag-Rab1
Arg-GlcNAcylated-Rab1

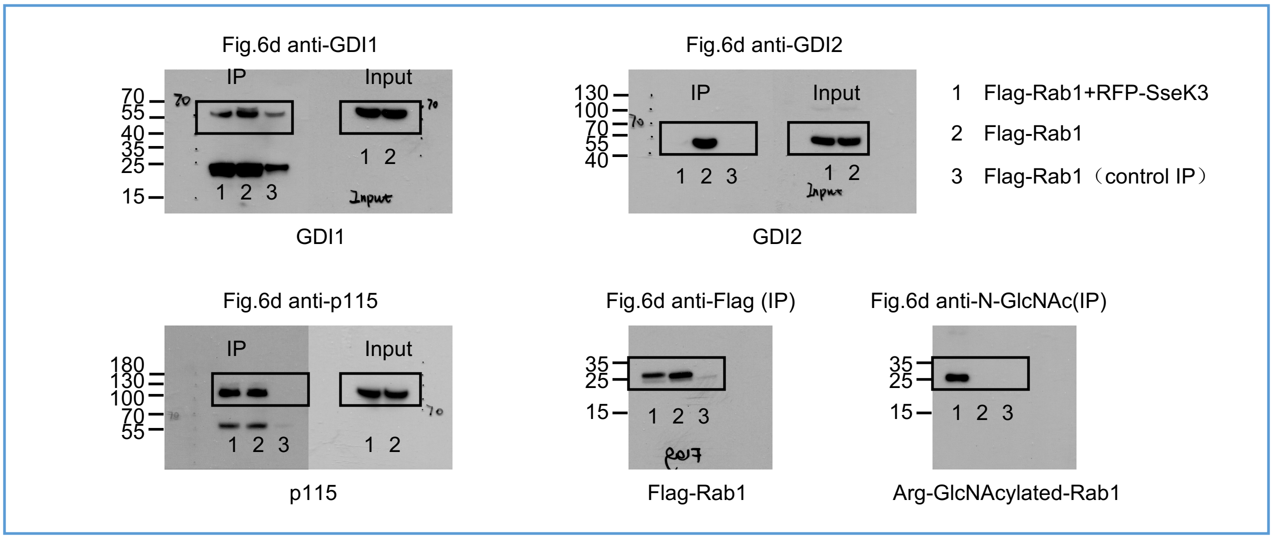
Fig.6d anti-GDI1
IP
Input
70
55
40
35
25
15
1 2 3
1 2
GDI1
Fig.6d anti-GDI2
IP
Input
130
100
70
55
40
1 2 3
1 2
GDI2
1 Flag-Rab1+RFP-SseK3
2 Flag-Rab1
3 Flag-Rab1（control IP）
Fig.6d anti-p115
IP
Input
180
130
100
70
55
1 2 3
1 2
p115
Fig.6d anti-Flag (IP)
35
25
15
1 2 3
Flag-Rab1
Fig.6d anti-N-GlcNAc(IP)
35
25
15
1 2 3
Arg-GlcNAcylated-Rab1

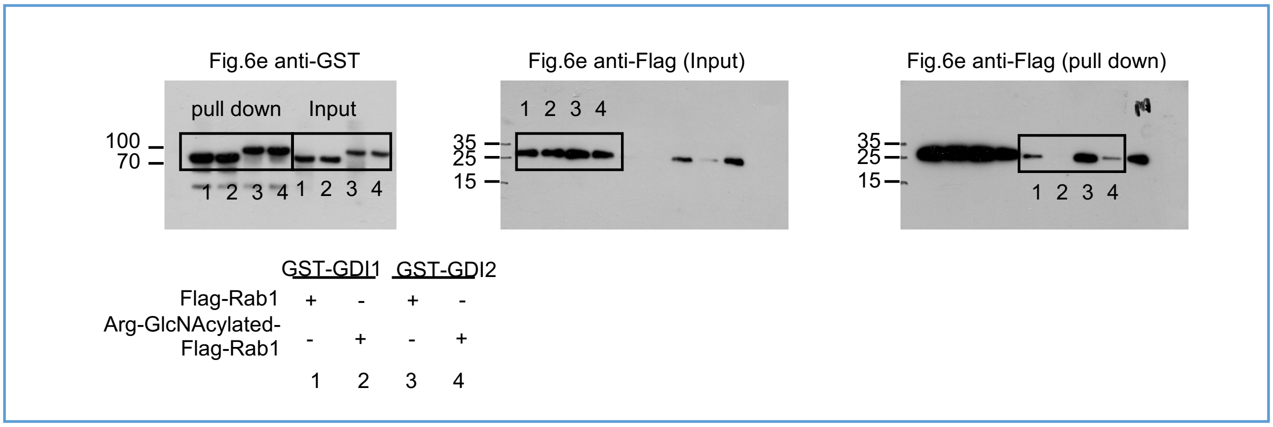
Fig.6e anti-GST
pull down
Input
100
70
1 2 3 4 1 2 3 4
Fig.6e anti-Flag (Input)
1 2 3 4
35
25
15
Fig.6e anti-Flag (pull down)
35
25
15
1 2 3 4
GST-GDI1
GST-GDI2
Flag-Rab1 + - + -
Arg-GlcNAcylated-Flag-Rab1 - + - +
1 2 3 4

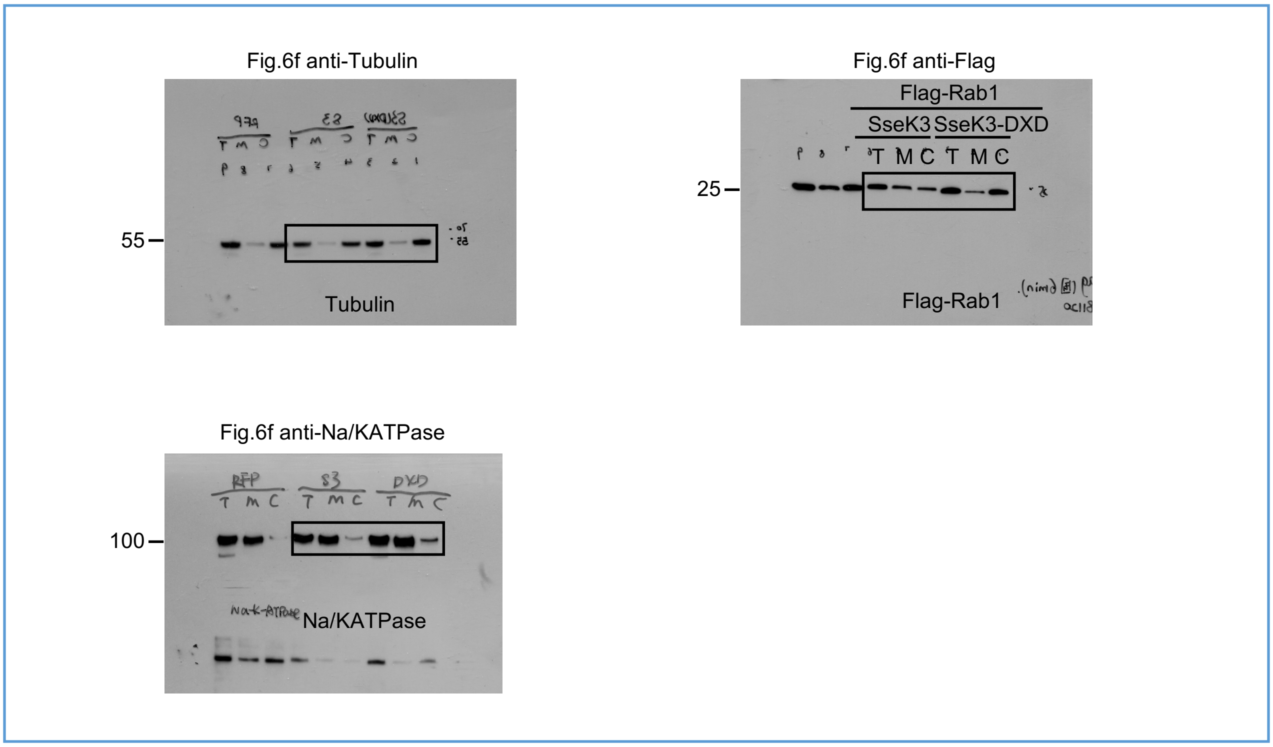
Fig.6f anti-Tubulin
55
Tubulin
Fig.6f anti-Flag
Flag-Rab1
SseK3
SseK3-DXD
T M C T M C
25
Flag-Rab1
Fig.6f anti-Na/KATPase
100
Na/KATPase

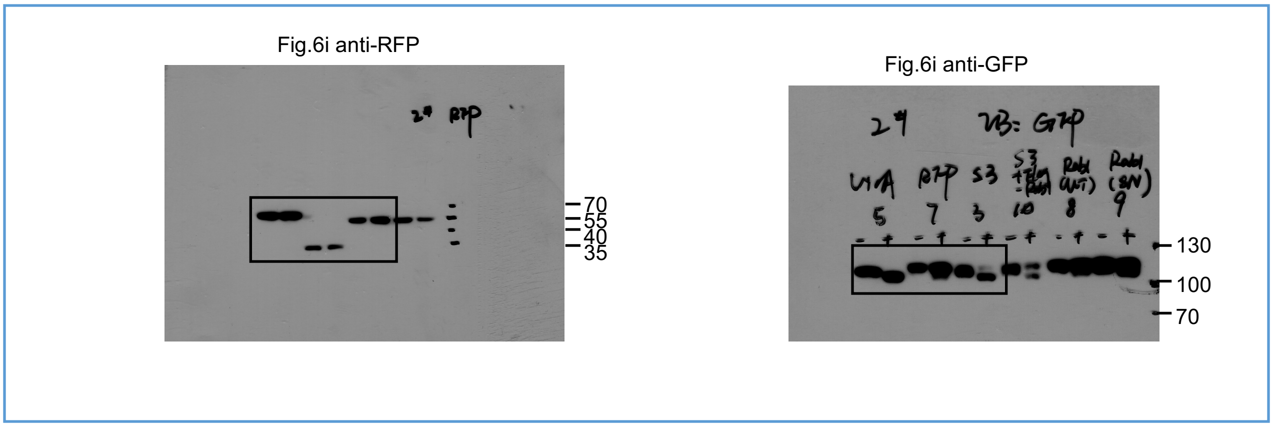
Fig.6i anti-RFP
70
55
40
35
Fig.6i anti-GFP
130
100
70

Supplementary Fig.6

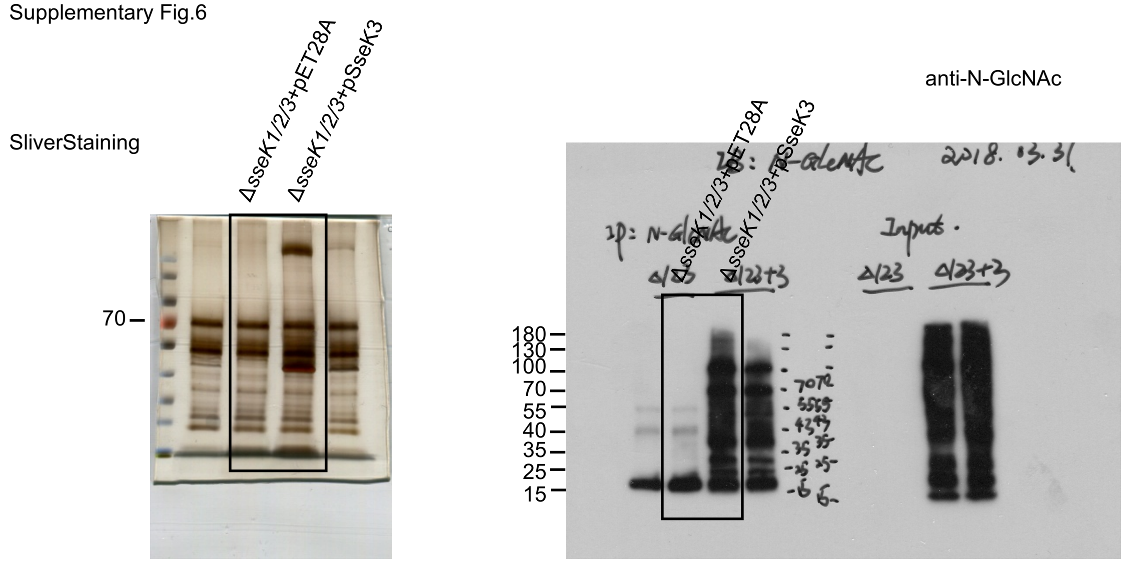

Supplementary Fig.7

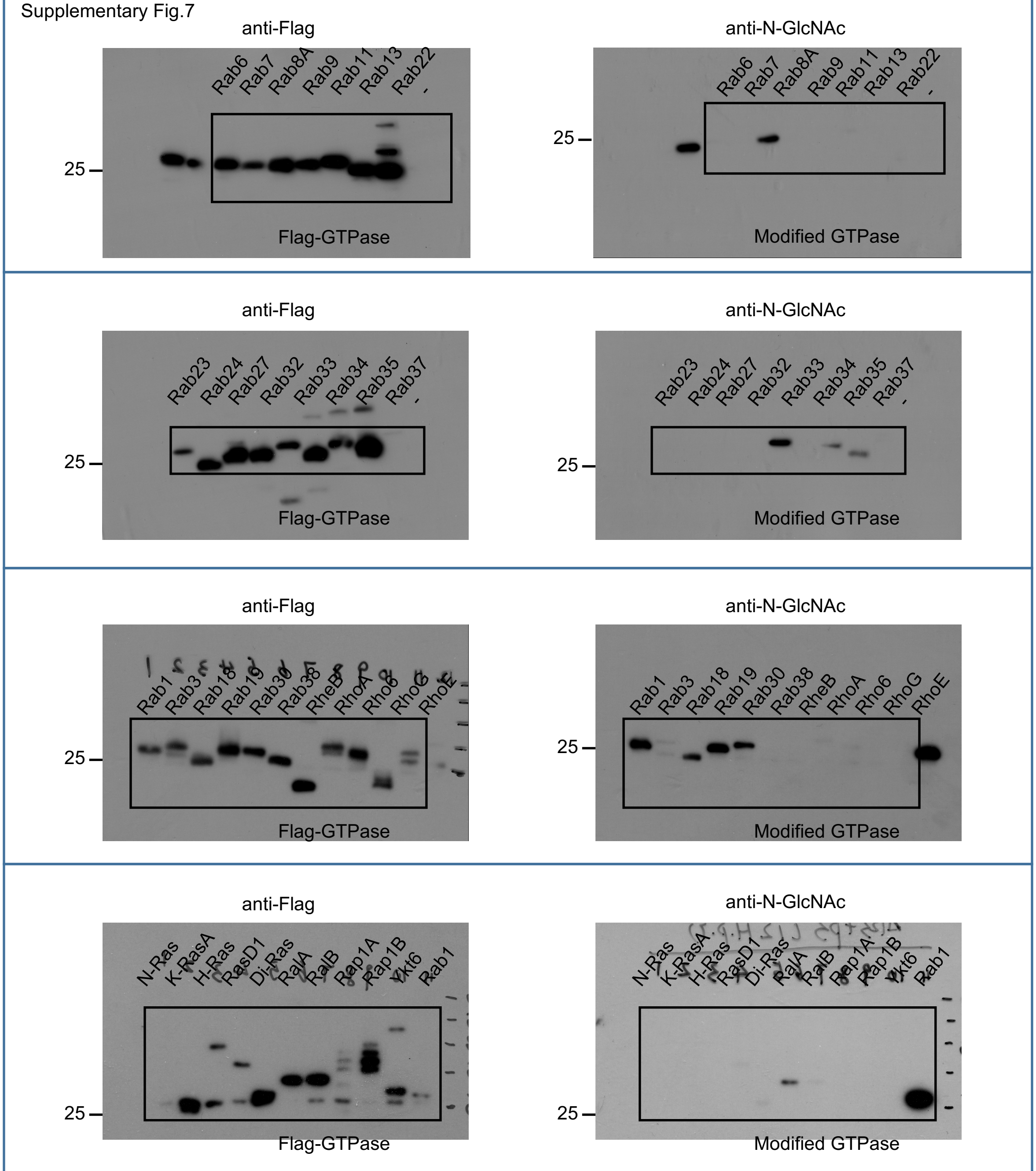

Supplementary Fig.14

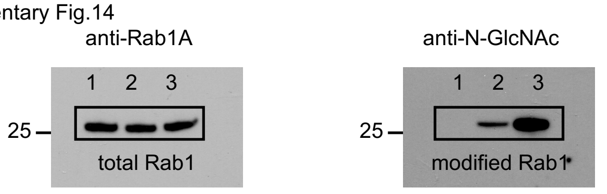

1 Rab1

2 mRab1

3 enriched mRab1

Supplementary Fig.16

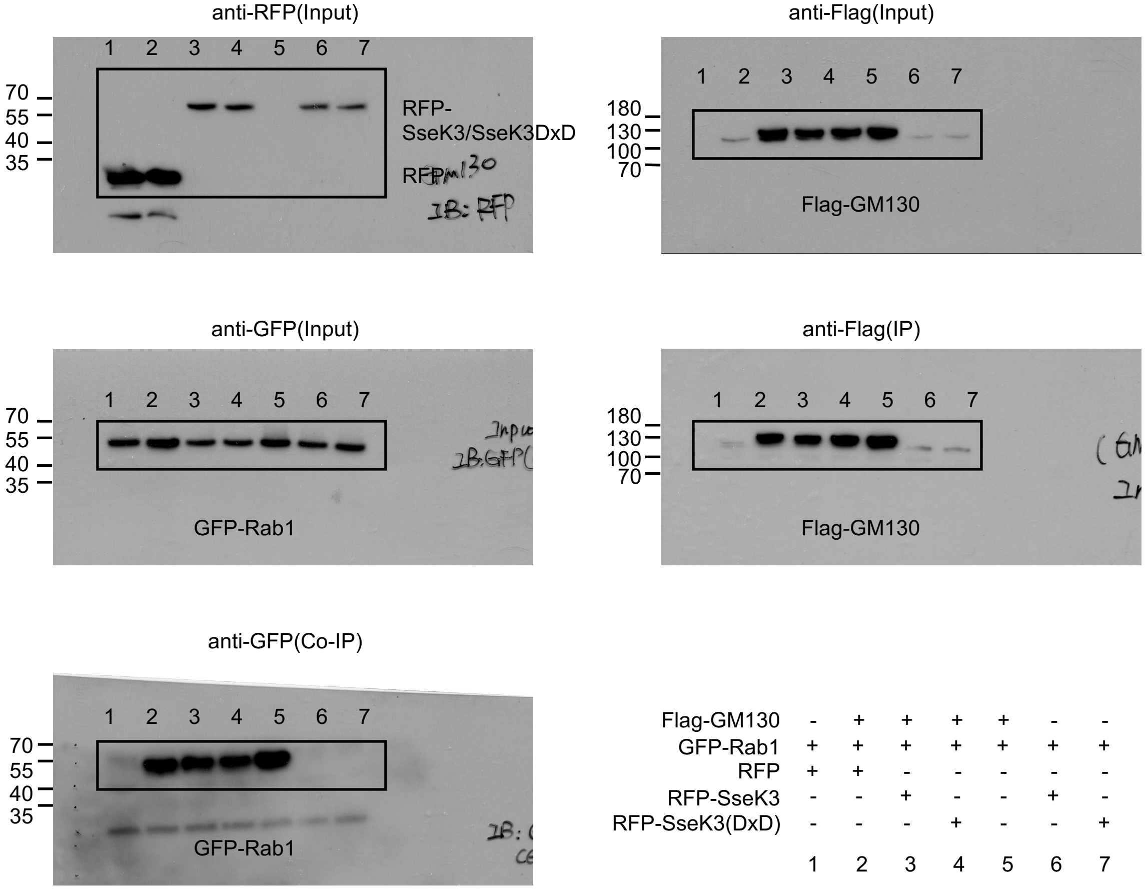

Flag-GM130	-	+	+	+	+	-	-
GFP-Rab1	+	+	+	+	+	+	+
RFP	+	+	-	-	-	-	-
RFP-SseK3	-	-	+	-	-	+	-
RFP-SseK3(DxD)	-	-	-	+	-	-	+
	1	2	3	4	5	6	7
