# Supplementary material for: Arginine GlcNAcylation of Rab small GTPases by the pathogen Salmonella Typhimurium
Source: Commun Biol. 2020 Jun 5;3:287. doi: 10.1038/s42003-020-1005-2 (PMC7275070; doi:10.1038/s42003-020-1005-2)
Supplement: Supplementary file 9 — Reporting Summary [file 42003_2020_1005_MOESM9_ESM.pdf]

nature research

Corresponding author(s): Shan Li

Last updated by author(s): May 8, 2020

Reporting Summary

Nature Research wishes to improve the reproducibility of the work that we publish. This form provides structure for consistency and transparency in reporting. For further information on Nature Research policies, see Authors & Referees and the Editorial Policy Checklist .

Statistics

For all statistical analyses, confirm that the following items are present in the figure legend, table legend, main text, or Methods section.

n/a	Confirmed	
☐	☒	The exact sample size (*n*) for each experimental group/condition, given as a discrete number and unit of measurement
☐	☒	A statement on whether measurements were taken from distinct samples or whether the same sample was measured repeatedly
☐	☒	The statistical test(s) used AND whether they are one- or two-sided<br>*Only common tests should be described solely by name; describe more complex techniques in the Methods section.*
☒	☐	A description of all covariates tested
☒	☐	A description of any assumptions or corrections, such as tests of normality and adjustment for multiple comparisons
☐	☒	A full description of the statistical parameters including central tendency (e.g. means) or other basic estimates (e.g. regression coefficient) AND variation (e.g. standard deviation) or associated estimates of uncertainty (e.g. confidence intervals)
☐	☒	For null hypothesis testing, the test statistic (e.g. *F*, *t*, *r*) with confidence intervals, effect sizes, degrees of freedom and *P* value noted<br>*Give P values as exact values whenever suitable.*
☒	☐	For Bayesian analysis, information on the choice of priors and Markov chain Monte Carlo settings
☒	☐	For hierarchical and complex designs, identification of the appropriate level for tests and full reporting of outcomes
☐	☒	Estimates of effect sizes (e.g. Cohen's *d*, Pearson's *r*), indicating how they were calculated

Our web collection on statistics for biologists contains articles on many of the points above.

Software and code

Policy information about availability of computer code

Data collection	Data collection by mass spectrometry was performed using Protein Deconvolution and Proteome Discoverer (version 2.2) softwares. Image J software (version 1.8.0) was used to calculate the Pearson correlation coefficient of co-localization in fluorescence images.
Data analysis	Data analysis was performed using Graphpad Prism (version 6).

For manuscripts utilizing custom algorithms or software that are central to the research but not yet described in published literature, software must be made available to editors/reviewers. We strongly encourage code deposition in a community repository (e.g. GitHub). See the Nature Research guidelines for submitting code & software for further information.

Data

Policy information about availability of data

All manuscripts must include a data availability statement. This statement should provide the following information, where applicable:

- Accession codes, unique identifiers, or web links for publicly available datasets
- A list of figures that have associated raw data
- A description of any restrictions on data availability

The MS raw data have been deposited to the iProx database (URL: https://www.iprox.org/page/PDV0141.html) and are available under the accession number IPX0002190000. The data will be released to public when the paper associated with this iProX ID is published. The protein sequences and plasmid sequencing results have been deposited in the figshare database (DOI: 10.6084/m9.figshare.12272705; https://figshare.com/s/78544f0f1453822fe202). Source data of gel and blot images are included in Supplementary Data1. All other relevant data supporting the findings of this study are available within the article and its Supplementary Information files and from the corresponding author upon reasonable request.

Field-specific reporting

Please select the one below that is the best fit for your research. If you are not sure, read the appropriate sections before making your selection.

- [x] Life sciences
- [ ] Behavioural & social sciences
- [ ] Ecological, evolutionary & environmental sciences

For a reference copy of the document with all sections, see nature.com/documents/nr-reporting-summary-flat.pdf

Life sciences study design

All studies must disclose on these points even when the disclosure is negative.

Sample size	No statistical methods were used to predetermine the sample sizes.
Data exclusions	No data were excluded.
Replication	The experimental findings were reliably reproduced. Please see details in the figure legends.
Randomization	No randomization was used in this study.
Blinding	No blinding was performed.

Reporting for specific materials, systems and methods

We require information from authors about some types of materials, experimental systems and methods used in many studies. Here, indicate whether each material, system or method listed is relevant to your study. If you are not sure if a list item applies to your research, read the appropriate section before selecting a response.

Materials & experimental systems

n/a	Involved in the study	
	x	Antibodies
	x	Eukaryotic cell lines
x		Palaeontology
	x	Animals and other organisms
x		Human research participants
x		Clinical data

Methods

n/a	Involved in the study	
x		ChIP-seq
x		Flow cytometry
x		MRI-based neuroimaging

Antibodies

Antibodies used

All antibodies are displayed in the following format:
Species, Antibody, Manufacturer, Catalog number,

Mouse, anti-α-tubulin, Sigma-Aldrich, Cat# T5168;
Rabbit, anti-β-actin, Sigma-Aldrich, Cat# A2066;
Rabbit, anti-Flag, Sigma-Aldrich, Cat# F7425;
Mouse, anti-Flag, Sigma-Aldrich, Cat# F1804;
Rabbit, anti-EGFP, Santa Cruz, Cat# sc-8334;
Rabbit, anti-RFP, MBL, Cat# PM005;
Mouse, anti-GST, Cell Signaling Technology, Cat# 2624S;
Mouse, anti-His Tag (HRP Conjugate), Cell Signaling Technology, Cat# 9991S;
Rabbit, anti-Rab1A, Cell Signaling Technology, Cat# 13075;
Mouse, anti-GM130, BD, Cat# 610822;
Mouse, anti-Human p230, BD, Cat# 611280;
Mouse, anti-HA Epitope Tag, BioLegend, Cat# 901501;
Rabbit, anti-Arg-GlcNAc, Abcam, Cat# ab195033;
Mouse, anti-DnaK, Abcam, Cat# ab69617;
Rabbit, Anti-Mannosidase II/MAN2A1, Abcam, Cat# ab12277;
Rabbit, Anti-LAMP1, Abcam, Cat# ab24170;
Mouse, Anti-Rab5A, Proteintech, Cat# 66339-1-Ig;

Validation

All antibodies were commercially available. All antibodies were validated for specific use and the relevant literature was listed on

the manufacturers' website.

Eukaryotic cell lines

Policy information about cell lines

Cell line source(s)	All cells are displayed in the following format:<br>Cell line, Organism, Tissue, Cell type, Source, Catalog number,<br>293T, Human, embryonic kidney, epithelial, ATCC, Cat# CRL-3216;<br>HeLa, Human, cervix, epithelial, ATCC, Cat# CCL-2;<br>RAW264.7, Mouse, Abelson murine leukemia virus–induced tumor, macrophage, ATCC, Cat# TIB-71;<br>MEF, Mouse, embryo fibroblast, fibroblast, ATCC, Cat# CRL-2991;<br>C57BL/6 mice-derived wild-type iBMDM cells from Feng Shao's lab (Shi J, et al., 2015).
Authentication	All cell lines used were authenticated by the manufacturers and checked morphologically under the microscope.
Mycoplasma contamination	All cell lines used were routinely tested for mycoplasma contamination and were mycoplasma free.
Commonly misidentified lines<br>(See ICLAC register)	No commonly misidentified cell lines were used.

Animals and other organisms

Policy information about studies involving animals; ARRIVE guidelines recommended for reporting animal research

Laboratory animals	Five- to six-week-old wild-type C57BL/6 mice were purchased from Liaoning Changsheng Biotechnology Co. and were maintained in the specific pathogen-free (SPF) facility at Huazhong Agricultural University.
Wild animals	This study did not involve wild animals.
Field-collected samples	This study did not involve samples collected from the field.
Ethics oversight	All animal experiments were carried out in accordance with the Ministry of Health national guidelines for housing and care of laboratory animals and performed in accordance with institutional regulations after review and approval by the Institutional Animal Care and Use Committee at Huazhong Agricultural University.

Note that full information on the approval of the study protocol must also be provided in the manuscript.
